# Liquid Biopsy in Organ Damage: small extracellular vesicle chip-based assessment of polytrauma

**DOI:** 10.3389/fimmu.2023.1279496

**Published:** 2023-11-15

**Authors:** Bingduo Wang, Aliona Wöhler, Johannes Greven, Rebekka J. S. Salzmann, Cindy M. Keller, Tobias Tertel, Qun Zhao, Ümit Mert, Klemens Horst, Ludmila Lupu, Markus Huber-Lang, Martijn van Griensven, Tom Erik Mollnes, Sebastian Schaaf, Robert Schwab, Christian P. Strassburg, Ingo G. H. Schmidt-Wolf, Bernd Giebel, Frank Hildebrand, Veronika Lukacs-Kornek, Arnulf G. Willms, Miroslaw T. Kornek

**Affiliations:** ^1^Department of Internal Medicine I, University Hospital Bonn of the Rheinische Friedrich-Wilhelms-University, Bonn, Germany; ^2^Department of General, Visceral and Thoracic Surgery, German Armed Forces Central Hospital, Koblenz, Germany; ^3^Department of Orthopaedics, Trauma and Reconstructive Surgery, University Hospital Rheinisch-Westfälische Technische Hochschule (RWTH) Aachen, Aachen, Germany; ^4^Institute for Transfusion Medicine, University Hospital Essen, University of Duisburg-Essen, Essen, Germany; ^5^Institute of Clinical and Experimental Trauma Immunology, University Hospital Ulm, Ulm, Germany; ^6^Department of Cell Biology-Inspired Tissue Engineering, MERLN Institute for Technology-Inspired Regenerative Medicine, Maastricht University, Maastricht, Netherlands; ^7^Research Laboratory, Nordland Hospital Bodø, Bodø, Norway; ^8^Department of Immunology, Oslo University Hospital, and University of Oslo, Oslo, Norway; ^9^Center of Molecular Inflammation Research, Norwegian University of Science and Technology, Trondheim, Norway; ^10^Department of Integrated Oncology, Center for Integrated Oncology, University Hospital Bonn of the Rheinische Friedrich-Wilhelms-University, Bonn, Germany; ^11^Institute of Molecular Medicine and Experimental Immunology, University Hospital Bonn of the Rheinische Friedrich-Wilhelms-University, Bonn, Germany; ^12^Department of General and Visceral Surgery, German Armed Forces Hospital, Hamburg, Germany

**Keywords:** monocytes, trauma, extracellular vesicles, exosomes, liquid biopsy, biomarker, triage

## Abstract

**Background:**

Despite major advances in medicine, blood-borne biomarkers are urgently needed to support decision-making, including polytrauma. Here, we assessed serum-derived extracellular vesicles (EVs) as potential markers of decision-making in polytrauma.

**Objective:**

Our Liquid Biopsy in Organ Damage (LiBOD) study aimed to differentiate polytrauma with organ injury from polytrauma without organ injury. We analysed of blood-borne small EVs at the individual level using a combination of immunocapture and high-resolution imaging.

**Methods:**

To this end, we isolated, purified, and characterized small EVs according to the latest Minimal Information for Studies of Extracellular Vesicles (MISEV) guidelines from human blood collected within 24 h post-trauma and validated our results using a porcine polytrauma model.

**Results:**

We found that small EVs derived from monocytes CD14^+^ and CD14^+^CD61^+^ were significantly elevated in polytrauma with organ damage. To be precise, our findings revealed that CD9^+^CD14^+^ and CD14^+^CD61^+^ small EVs exhibited superior performance compared to CD9^+^CD61^+^ small EVs in accurately indicating polytrauma with organ damage, reaching a sensitivity and a specificity of 0.81% and 0.97%, respectively. The results in humans were confirmed in an independent porcine model of polytrauma.

**Conclusion:**

These findings suggest that these specific types of small EVs may serve as valuable, non-invasive, and objective biomarkers for assessing and monitoring the severity of polytrauma and associated organ damage.

## Background

Blood-borne biomarkers are the simplest and most non-invasive source of biomarkers for all types of diseases, including severe COVID-19 and cancer (1, 2). In the last decade, many exciting biomarkers have been explored, discovered, or validated for clinical use as detectable biomarkers in blood. Among these biomarkers, such as circulating cell-free DNA (ccfDNA), circular RNAs (circRNAs), circulating tumour cells (CTCs), and soluble proteins, extracellular vesicles (EVs) have recently received the most attention (2–5). EVs are vesicles that are naturally released from cells. They consist of a lipid bilayer, lack a functional nucleus, and cannot proliferate. In addition, EVs are classified according to their biogenesis and cellular origin (6, 7). The “Minimal Information for Studies of Extracellular Vesicles 2018” (MISEV2018) includes caveats and protocols and defines small EVs as fractions that may consist of EVs derived from late endosomes (8). EVs are released under steady-state conditions and secreted by activated and apoptotic cells (9).

EVs have been shown to be beneficial for cancer screening in pancreatic carcinoma (10), biliary carcinomas in the gallbladder and cholangiocarcinoma (11), other cancer entities such as hepatocellular carcinoma (12), brain tumours (13), and other non-malignant diseases (14). However, the EV methodology is not limited to cancer screening, and few research groups have explored the potential of EVs in polytrauma and trauma care (15, 16), leading to the conclusion that small EVs might also be beneficial. Recently, it was reported that small EVs may be attributed to a higher mortality rate in polytrauma with haemorrhagic shock (17) and altered platelet levels (18).

Polytrauma remains one of the leading causes of death worldwide and the leading cause of disability in patients younger than 35 years (19). However, classifying potentially severely injured patients in prehospital care and objectively classifying and quantifying the complexity and quality of trauma remain problematic Algorithmic and objective data-based strategies are needed when decisions must be made when medical resources are scarce, not only in triage situations (20, 21). Only limited information about visible and recognizable damage can be obtained during the initial assessment of patients at the scene, in the emergency department, or during transport until maximum care is provided. Novel blood-borne assessment methods based on liquid biopsy could be supportive and objectively provide the required information.

The aim of our Liquid Biopsy in Organ Damage (LiBOD) study was to investigate whether small EVs are altered in polytrauma, including internal organ injury, such as rupture of parenchymal organs, in humans and a porcine polytrauma model. The physiological response to trauma is a complex process, as outlined by Lord et al., who summarized the inflammatory cascades triggered by trauma (22). Following traumatic injury, there is an increase in the frequency of the classic inflammatory monocyte subset in humans, characterized as CD14^+^CD16^−^, post-injury (23). We employed the ExoView^®^ Reader R100 to immobilize small EVs and distinguish between different subpopulations of monocytes and platelets, including megakaryocytes, aiming to assess their clinical performance in both human polytrauma (injury severity score (ISS) ≥ 15) and porcine polytrauma models. Our findings revealed that CD9^+^CD14^+^ small EVs derived from monocytes exhibited superior performance compared to CD9^+^CD61^+^ small EVs in accurately indicating polytrauma with organ damage.

## Methods

### Ethics: human specimens

This study was developed as a non-interventional and retrospective study according to the Clinical Trials Directive (EU) 2001/20/EC and Clinical Trials Regulation (EU) NO 536/2014. The Ethics Committee of the responsible State Chambers of Medicine in Rhineland-Palatinate, Germany (ANr.: 2020-15050) approved the human study part. Informed consent was obtained from all the patients or their legal representatives. The presented data are part of a study that has been registered on the International Clinical Trials Registry Platform through the German Registry for Clinical Trials (DRKS 00026025; https://drks.de/search/en/trial/DRKS00026025).

### Human study cohort

**Figure 1:**
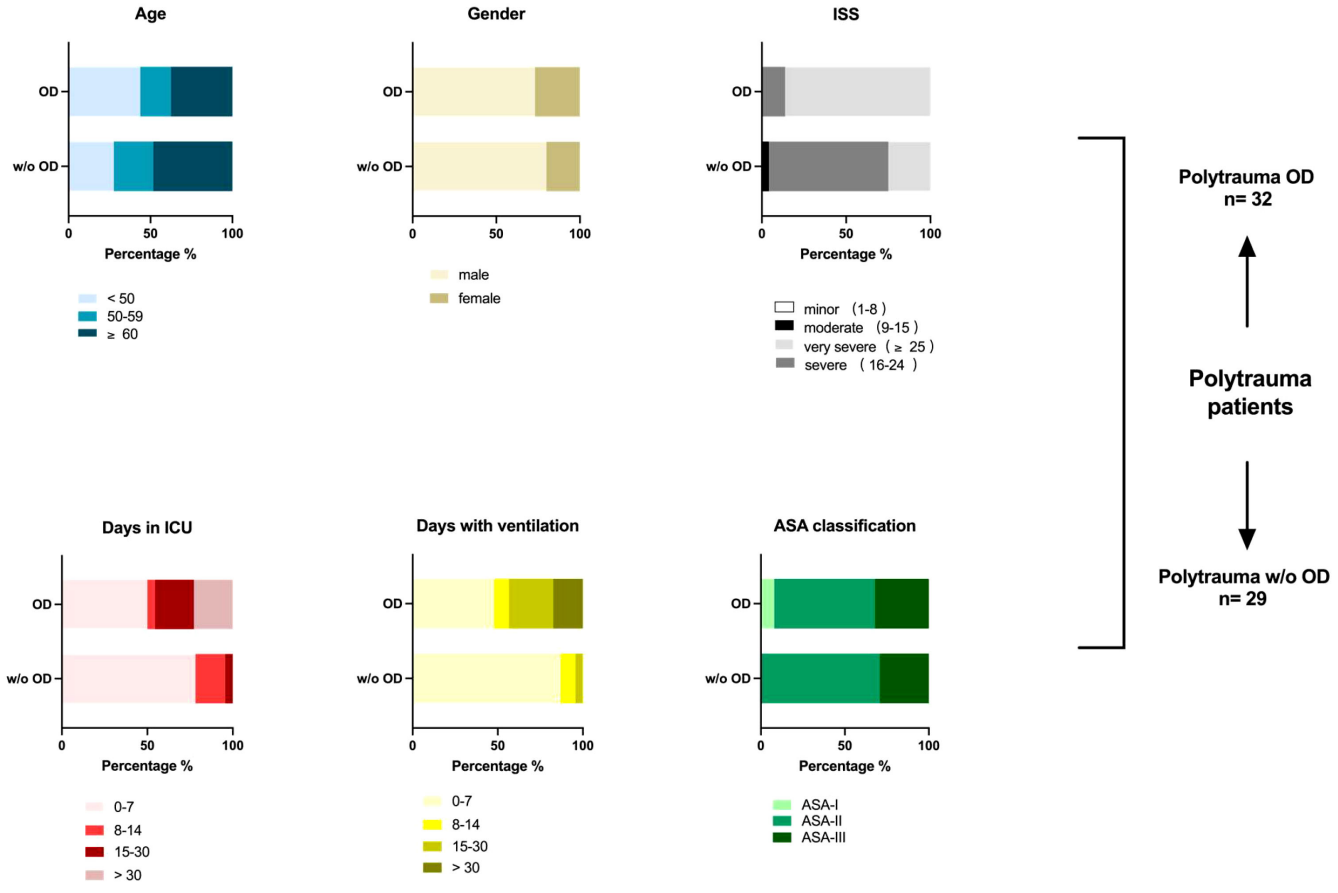
Demographics of polytrauma (ISS ≥ 15) patients. Overview of patient’s parameters post-trauma, including ISS score, days in ICU, days with invasive ventilation, and ASA classification (if available), in addition to age and gender distribution. A total of 61 polytrauma patients with internal organ damage (polytrauma OD, n = 32) and polytrauma patients without internal organ damage (polytrauma w/o OD, n = 29) were included in this human study in agreement with the given regulations of the responsible State Chambers of Medicine in Rhineland-Palatinate, Germany (ANr.: 2020-15050). Polytrauma OD includes subgroups such as lacerated parenchymal internal organs such as the liver and spleen, kidney rupture, severe lung contusion, vascular dissection of the aorta, and amputation. Amputation was added to the internal organ damage group due to its severity and strain on the amputee regarding blood loss and pathogen infiltration. For further details, see **Supplementary Table 1**. ISS, injury severity score; ICU, intensive care unit; ASA, American Society of Anesthesiologists.

Polytrauma patients were included if they had injury severity scores (ISS) ≥ 15 and i) penetrating injuries to the torso-cervical region, ii) fell from a height of more than 1.5 m, iii) had a traffic accident with frontal impact or intrusion of more than 50–75 cm or change in speed of delta > 30 km/h, and iv) had a two-wheeler collision, such as in a motorcycle, bicycle, e-bike, or e-scooter. Typically, polytrauma patients were excluded when i) there were any doubts about the patient’s ability to provide consent or their legal representatives had signs of dementia, ii) patients were younger than 18 years, or iii) they were pregnant. The general characteristics of the patients are summarized in **Supplementary Table 1** and **Figure 1**. Additionally, blood parameters such as erythrocyte, thrombocyte, and leucocyte levels, in addition to medications and drugs given after admission, are summarized in **Supplementary Figure 1**. Inclusion and exclusion criteria were set according to the Level 3 guidelines on the treatment of patients with severe or multiple injuries (Association of the Scientific Medical Societies, AWMF Register-Nr. 012/019 - Polytrauma Guideline Update Group).

### Human cell lines

For details, please see **Supplement Material** and Methods.

### Differentiation and stimulation of THP-1 monocyte cells *in vitro*

Please see **Supplement Material** and Methods.

### Cell surface staining for flow cytometric analysis of THP-1 human cell lines

Please see **Supplement Material** and Methods.

### Isolation and purification of THP-1-derived small EVs by size exclusion chromatography and ultrafiltration from cell culture supernatant

Please see **Supplement Material** and Methods.

### Isolation of THP-1-derived large EVs by centrifugation from cell culture supernatant

Please see **Supplement Material** and Methods.

### Isolation of serum from human blood

Please see **Supplement Material** and Methods.

### Isolation of small EVs from human serum

Human serum (1 mL) was centrifuged at 10,000 × *g* for 10 min to remove lipemia and most of the large EVs. EV-containing supernatant was filled up to 2 mL with filtered phosphate-buffered saline (PBS) (pH 7.4, 0.22 µm). Diluted and precleared serum (2 mL) was loaded onto a qEV2/70 size exclusion chromatography (SEC) column, and a buffer flow volume of 14.1 mL was discarded, after which five fractions (2 mL each) were collected. These five fractions (each 2 mL) were pooled together and concentrated to 1 mL of the large EV-depleted small EV fraction with a 3-kDa molecular weight cut-off (MWCO) filter for the ExoView^®^ Reader-based study.

### Isolation and purification of small EVs from pig plasma

Porcine plasma (1 mL) was centrifuged at 2,500 × *g* for 15 min at room temperature (RT). The supernatant was filled to 2 mL with filtered PBS (pH 7.4, 0.22 µm). Diluted and precleared porcine plasma (2 mL) was loaded onto a qEV2/70 SEC column. A buffer flow volume of 14.1 mL was discarded, and five fractions (2 mL each) were collected. These five fractions were pooled and concentrated to 1 mL using a 3-kDa MWCO filter for the ExoView^®^ Reader-based study.

### Transmission electron microscopy of EVs

Please see **Supplement Material** and Methods.

### Protein isolation and quantification from small EVs

Please see **Supplement Material** and Methods.

### Western blotting analysis

Please see **Supplement Material** and Methods.

### Nanoparticle tracking analysis of small EVs

Please see **Supplement Material** and Methods.

### Small EV quantification

Isolated and purified human and porcine small EV samples were characterized by a single-particle interferometric reflectance imaging sensor (SP-IRIS) using the ExoView^®^ R100 Reader (Unchained Labs, Boston, MA, USA; previously NanoView^®^ Biosciences, Boston, MA, USA). According to the ExoView^®^ kit assay protocol (v380.6, revised August 2021), the human ExoView^®^ Tetraspanin Chip (Unchained Labs, Boston, MA, USA; previously NanoView^®^ Biosciences, Boston, MA, USA, Product No.: EV-TETRA-C) was pre-scanned to detect debris before loading the samples. The human ExoView^®^ Tetraspanin Chips were precoated with anti-CD81, anti-CD63, and anti-CD9 EV capture antibodies and MIgG capture controls to ensure optimal performance.

Small EV samples were diluted in 1 × incubation solution I and immobilized on a human ExoView^®^ Tetraspanin Chip overnight (18 h) at RT. After incubation, the human ExoView^®^ Tetraspanin Chips were washed three times in 1 × solution A, followed by incubation with anti-human tetraspanin antibodies such as anti-CD9, anti-CD63, and anti-CD81 (**Supplementary Table 4**) for 1 h. The ExoView^®^ Tetraspanin Chips were then washed in 1 × solution B, dried, and imaged using ExoView^®^ R100. The acquired data were analysed using ExoView Analyzer (Version 3.1.4) (24).

For quantification of porcine-derived small EVs, antibody-uncoated ExoFlex^®^ chips were coated with commercially available anti-pig CD9 antibody (**Supplementary Table 4**) according to the manufacturer’s recommendations. Briefly, CD9 monoclonal antibody (MA1-80307, Invitrogen, Thermo Fisher, Waltham, MA, USA) was conjugated with linker 1 and incubated on ExoFlex^®^ chips. ExoFlex^®^ chips with immobilized CD9^+^ small EVs were incubated with anti-pig CD14 fluorescein isothiocyanate (FITC) (MA5-28286, Invitrogen) for 1 h, washed in solution B, dried, and imaged using ExoView^®^ R100. The acquired data were analysed using the ExoView Analyzer.

Fluorescent cut-offs were set relative to the MIgG control: anti-human CD14 APC in the CF647 channel, 450; anti-human CD61 PE in the CF555 channel, 300; and anti-pig CD14 FITC in the CF488 channel, 450. All the anti-human and anti-porcine antibodies used are summarized in **Supplementary Table 4**.

### Ethics: porcine study

This study was approved by the responsible State Agency for Nature, Environment, and Consumer Protection of North Rhine-Westphalia (Landesamt für Natur, Umwelt und Verbraucherschutz Nordrhein-Westfalen AZ: 81-02.04.2020. A215). Furthermore, a porcine study was designed considering the German legislation governing animal studies, which followed the Principles of Laboratory Animal Care (25). Additionally, all porcine experiments of this study adhered to the ARRIVE Guidelines for reporting animal research (26).

### Animals: porcine study

The porcine model was previously established by the Department of Orthopedics, Trauma, and Reconstructive Surgery at the University Hospital RWTH Aachen and has been published elsewhere (27–29). The results of this study were part of a larger study design that included an additional group [for additional subgroup information, see Lupu et al. (29)]. In total, 22 male pigs [German Landrace (*Sus scrofa*)] weighing 35 ± 5 kg were used for multiple trauma induction experiments. All pigs underwent an initial clinical examination, were acclimated to a new environment for 7 days prior to operation/trauma induction, and were housed in ventilated rooms with food and water *ad libitum*.

### Multiple trauma model: porcine study

#### a) Instrumentation and anaesthesia

Please see **Supplement Material** and Methods.

#### b) Trauma and haemorrhage

Please see **Supplement Material** and Methods.

#### c) Group allocation

Please see **Supplement Material** and Methods.

### Plasma isolation from full-blood porcine study

Please see **Supplement Material** and Methods.

### Statistical analysis

All data are presented as medians with a 95% confidence interval (CI). A one-way ANOVA was used to analyse differences among the three experimental groups, followed by a *post-hoc* test. Fisher’s least significant difference (LSD) test was applied for multiple comparisons of subgroups when one-way ANOVA was positive, and Bartlett’s test for equal variance was successful. The differences between two independent experimental subsets were determined using a two-tailed, unpaired t-test. The area under the receiver operating characteristic curve (AUROC), sensitivity, specificity, and associated cut-off values were calculated. Pearson’s correlation coefficient was calculated to measure the linear correlations between the two sets of data. Statistical results were considered significant if the *p*-value was <0.05. The experimental strength was calculated *post-hoc*.

Fluorescence-activated cell sorting (FACS) data were analysed using FlowJo 10 for MAC OSX (Tree Star Inc., Ashland, OR, USA). ExoView data were processed using the ExoView^®^ Analyzer (Version 3.1.4, Unchained Labs). Statistical analysis was performed using GraphPad Prism 9 (GraphPad Software Inc., La Jolla, USA) and the G*Power program (version 3.1.9.2, Düsseldorf, Germany). Figures were created using GraphPad Prism version 9. Some figures were done with the help of BioRender.com.

## Results

### Antibody validation on cultured THP-1 cells for EV detection

**Figure 2:**
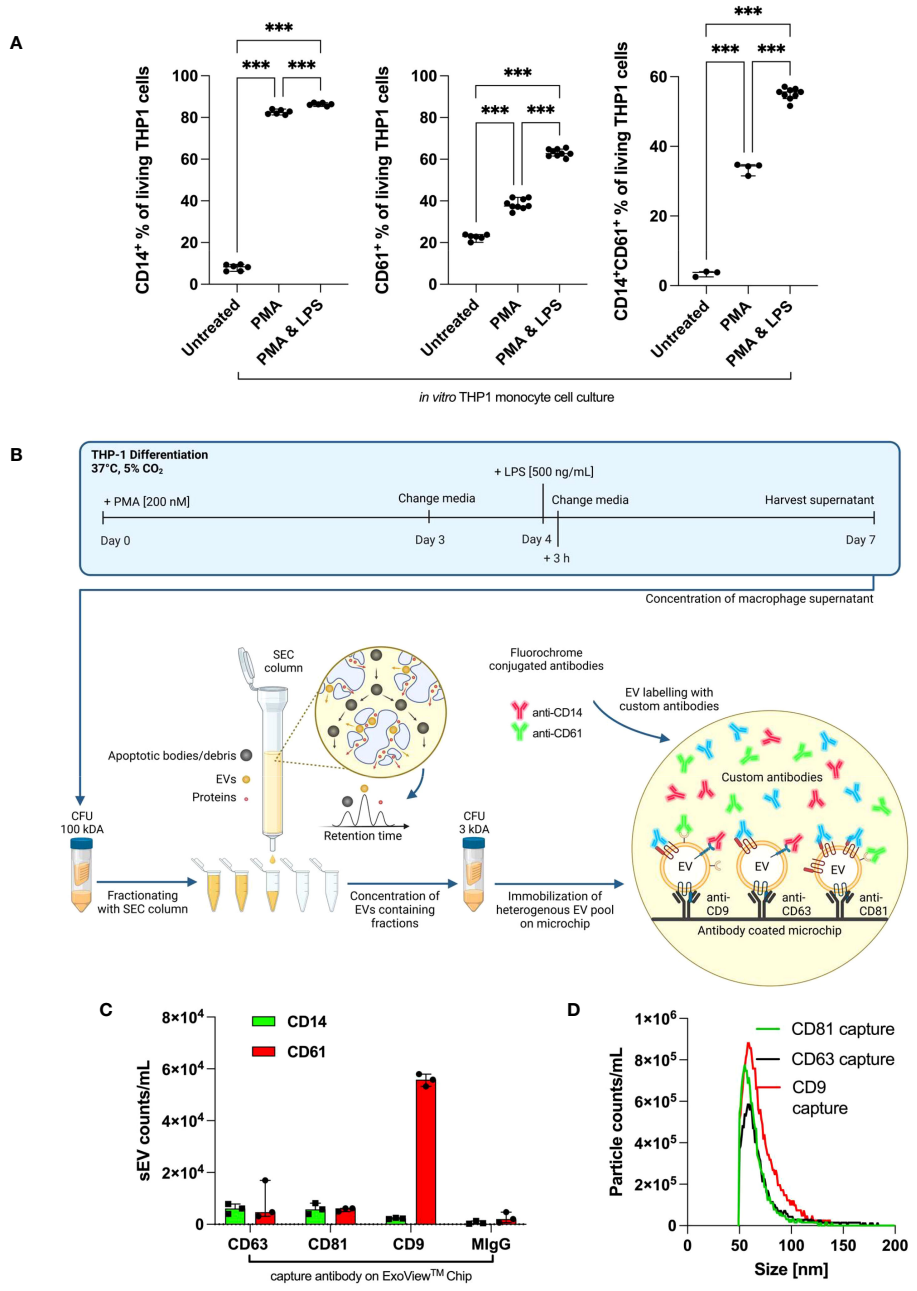
Antibody validation on cultured THP-1 cells and THP1-derived small EVs. **(A)** *In vitro*, human THP-1 monocyte cells were treated with PMA alone or PMA/LPS (each n ≥ 3). Values are given as mean with SEM. Column statistical analysis performed by one-way ANOVA test including Fisher’s LSD *post-hoc* test for multiple comparisons (*p < 0.05; **p < 0.01; ***p < 0.001; CD14^+^: F = 9325, df = 2, CD61^+^: F = 754.7, df = 2; CD14^+^CD61^+^: F = 1337, df = 2). **(B)** Overview of small EV generation, SEC isolation, and the use of the ExoView^®^ Tetraspanin kit. Typically, THP-1 was stimulated with 200 nM of PMA and 500 ng/mL of LPS to complete a cell type-specific phenotypical changing cycle within 1 week. **(C)** EV counts as immobilized by CD9, CD63, and CD81 capture antibodies and subsequently stained with anti-CD14 and anti-CD61 on the ExoView^®^ Tetraspanin microchip. **(D)** EV size distributions for particles as immobilized by CD9, CD63, and CD81 capture antibodies on the ExoView^®^ Tetraspanin microchip. CFU, centrifugal filter unit; EVs, extracellular vesicles; PMA, phorbol 12-myristate 13-acetate; LPS, lipopolysaccharide; LSD, least significant difference; SEC, size exclusion chromatography.

First, we confirmed that non-stimulated THP-1 cells lacked CD14 expression (**Figure 2A**) (30). Stimulation with phorbol 12-myristate 13-acetate (PMA) or PMA/lipopolysaccharide (LPS) is required to markedly increase CD14 expression. For CD61 and CD14^+^CD61^+^ cells, a similar upregulation was achieved with PMA/LPS. Therefore, THP-1 cells are suitable for the evaluation of anti-CD14 and anti-CD61 antibody testing of THP-1-derived EVs *in vitro*.

### THP-1 cell culture-derived small EVs and their tetraspanin expression

Per the ExoView^®^ Tetraspanin Chip, 8.5 × 10^11^ EVs derived from THP-1 cells after SEC and ultrafiltration (UF) purification were used. The EV yield and purity were evaluated *via* nanoparticle tracking analysis (NTA) measurements, allowing us to load the ExoView^®^ Tetraspanin Chips with the same small EV numbers. Small EVs were captured/immobilized on the chip using CD9, CD63, and CD81. Immobilized small EVs were distributed among CD63, CD81, and CD9 capture antibodies and designated as isotype control MIgG. The whole small EV isolation and analysis procedure including THP-1 cultering is graphically summarised in **Figure 2B**.

The amount of immobilized small EVs captured by the ExoView^®^ Tetraspanin Chip was set to 100%, and the distribution of small EVs was calculated among designated capture antibody spots: anti-CD63 24,9%, CD81 13%, and CD9 62% (**Figure 2C**). The captured small EVs were stained for CD14 and CD61. Accordingly, a relatively low number of CD9^+^CD14^+^ small EVs was observed compared with CD63^+^CD14^+^ and CD81^+^CD14^+^ EVs. In the case of CD61, CD9^+^CD61^+^ small EVs were predominant over CD63^+^CD61^+^ and CD81CD61^+^ small EVs. The associated representative spot pictures can be found in supplementary (**Supplementary Figure 2**). Importantly, these data unequivocally demonstrated that our chosen antibodies, anti-CD14, and anti-CD61, were able to detect CD14 and CD61 surface antigens located on small EVs derived from human monocytes/macrophages. **Figure 2D** depicts the median EV diameters as captured by the CD9/CD63/CD81 probes on the ExoView^®^ Tetraspanin Chip.

We used Western blotting to characterize isolated small EVs based on their protein content, in particular, plasma membrane-associated tetraspanin CD63, secreted calreticulin, cytosolic protein TSG101, and housekeeping protein β-actin as a loading control (see **Supplementary Figure 3**). In addition to negative calreticulin, CD63, and TSG101 were positive in our isolated small EVs. β-Actin served as a loading control. The preparation of large EVs was run as a control, which led to no detectable signals for TSG101 and calreticulin. CD63 is faint in large EVs and typically enriched in small EVs.

### Human serum-derived small EVs are predominantly CD63- and CD9-positive

**Figure 3:**
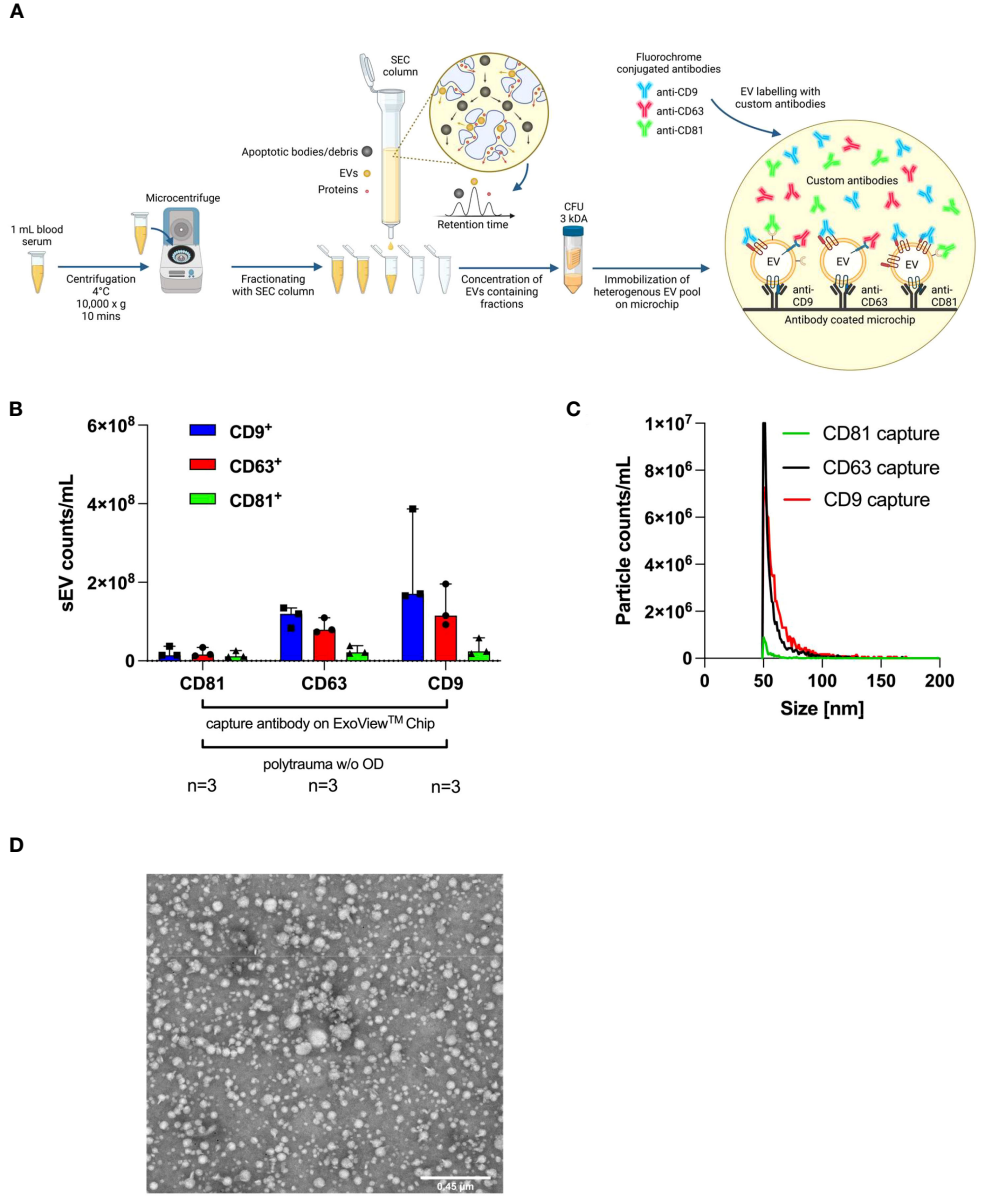
Characterisation of small EVs isolated from polytrauma patients’ serum samples by surface antigens, size, and TEM. ExoView^®^ Reader 100 is capable of determining the diameter of individual captured small EVs binding to a precoated ExoView^®^ Tetraspanin Chip with anti-CD9, anti-CD63, and anti-CD81 on designated capture spots by manufacture. **(A)** Workflow SOP for isolation from 1 mL of human serum collected from polytrauma patients (ISS ≥ 15), including isolation *via* SEC and UF and a final interferometric detection system with the ExoView^®^ Reader 100-based methodology. Manufacturer-supplied antibodies, anti-CD9 (CF488A), anti-CD63 (CF647), and anti-CD81 (CF555) were used for the detection of indicated tetraspanins on captured small EVs. **(B)** Tetraspanin distribution on CD9^+^, CD63^+^, or CD81^+^ captured small EVs on the ExoView^®^ Tetraspanin Chip (n= 3). The graph depicts the median with a 95% confidence interval (CI). **(C)** Sizes of EVs as immobilized with CD9^+^, CD63^+^, or CD81^+^ on ExoView^®^ Tetraspanin Microchips. **(D)** Representative transmission electron microscopy (TEM) picture of SEC-isolated small EVs demonstrating small EV sizes ranging from 50 nm to 200 nm. CFU, centrifugal filter unit; EVs, extracellular vesicles; SOP, standard operating procedure; ISS, injury severity score; SEC, size exclusion chromatography; UF, ultrafiltration.

**Figure 3A** depicts our standard operating procedure (SOP) for the isolation of small EVs, starting with 1 mL of human serum. Isolated human serum-derived small EVs were characterized by the ExoView^®^ Tetraspanin Chip using anti-CD9, anti-CD63, and anti-CD81 as custom antibodies for exact quantification of small EV subpopulations (CD9^+^, CD63^+^, and CD81^+^ small EVs). The immobilized small EVs were mostly CD9 and CD63 positive, and a small population was CD81 positive (**Figure 3B**). ExoView^®^ SP-IRIS measurements demonstrated that the size of each captured small EV ranged from 50 nm to 70 nm in diameter (**Figure 3C**). Accordingly, transmission electron microscopy (TEM; **Figure 3D**) was used to detect EVs within a size range measured on the chip. Isolated small EVs showed typical morphology and size for EVs, as previously published (24).

### EV subpopulations show unique distribution in polytrauma patients

**Figure 4:**
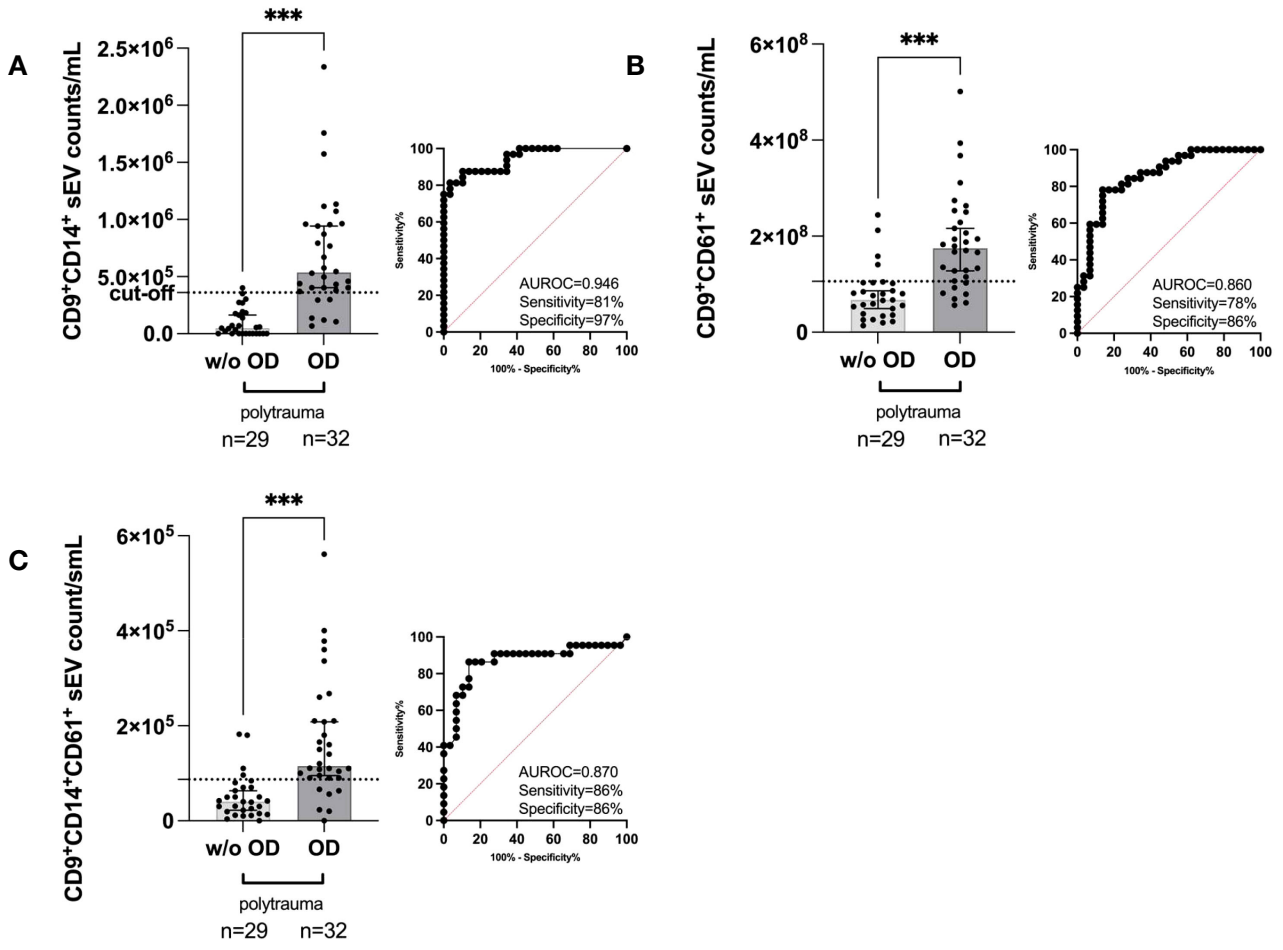
Quantification of the indicated human serum-derived small EV subpopulation as defined by the small EV marker CD9 plus custom anti-CD14 and anti-CD61 on ExoView^®^ Tetraspanin Chips. **(A)** CD9^+^CD14^+^ small EV counts in polytrauma (ISS ≥ 15, within 24 h post-trauma) with internal organ damage (polytrauma OD) *vs.* polytrauma without internal organ damage (polytrauma w/o OD). **(B)** CD9^+^CD61^+^ small EV counts in small EVs in polytrauma OD *vs.* polytrauma w/o OD. **(C)** CD9^+^CD14^+^CD61^+^ small EV counts in small EVs in polytrauma OD *vs.* polytrauma w/o OD. Statistical significance was assessed by a two-tailed, unpaired t-test (*p < 0.05; **p < 0.01; ***p < 0.001; **(A)** t = 6.248, df = 59; **(B)** t = 5.100, df = 59; **(C)** t = 4.679, df = 59). All these scatter dot plots show lines at the median with a 95% CI. *Post-hoc* calculated power (1 − β err prob) is 1.0. Including corresponding AUROC, sensitivity, specificity, and cut-off values. CFU, centrifugal filter unit; EV, extracellular vesicle; ISS, injury severity score; AUROC, area under the receiver operating characteristic curve.

Anti-CD9, anti-CD63, and anti-CD81 precoated ExoView^®^ Tetraspanin Chips allow quantification of the individual numbers of the three small EV subpopulations as defined by CD9/CD63/CD81 (31). Among all investigated small EV subpopulations and our additional antibodies targeting CD14 and CD61, the following subpopulations could be distinguished: CD9^+^CD14^+^ small EVs, CD9^+^CD61^+^ small EVs, and double-positive CD9^+^CD14^+^CD61^+^ small EVs. All three types of small EV subpopulations exhibited a higher median count [CD9^+^CD14^+^ small EVs (**Figure 4A**), CD9^+^CD61^+^ small EVs (**Figure 4B**), and double-positive CD9^+^CD14^+^CD61^+^ small EVs (**Figure 4C**)] and were capable of distinguishing patients with polytrauma organ damage (OD) from patients without polytrauma (polytrauma without OD). Representative ExoView^®^ Tetraspanin capture spots are depicted in **Supplementary Figure 4**. Accordingly, CD9^+^CD14^+^ small EVs in polytrauma OD (median, 5.372 × 10^5^/mL serum) were significantly increased by 11.2-fold (*p* < 0.001) compared to those in polytrauma without OD (median, 48.0 × 10^4^/mL serum) with a calculated cut-off of 3.6 × 10^5^/mL serum. The associated clinical performance was as follows: AUROC = 0.9461, sensitivity = 81.25%, and specificity = 96.55% (**Figure 4A**). CD9^+^CD61^+^ small EVs (median, 1.747375 × 10^8^/mL serum, **Figure 4B**) and CD9^+^CD61^+^CD14^+^ small EVs (median, 1.1525 × 10^5^/mL serum, **Figure 4C**) were 2.6-fold (*p* < 0.001) and 2.9-fold (*p* < 0.001) elevated compared to polytrauma without OD, respectively (polytrauma w/o OD: CD9^+^CD61^+^ small EVs = 6.74935 × 10^7^/mL serum and CD9^+^CD14^+^CD61^+^ small EVs = 4.0 × 10^4^/mL serum). Associated cut-offs were set for CD9^+^CD61^+^ small EVs at 1.05860505 × 10^8^/mL serum with an AUROC of 0.8599, sensitivity of 78.13%, and specificity of 86.21%; for CD9^+^CD14^+^CD61^+^ small EVs, the cut-off was 8.7 × 10^4^/mL serum, AUROC of 0.8699, sensitivity of 86.36%, and specificity of 86.21%. All data for the individual small EV populations and subpopulations are summarized in **Supplementary Table 5**.

### The composition of small EV subpopulations follows the 7-day recovery period for polytrauma patients

**Figure 5:**
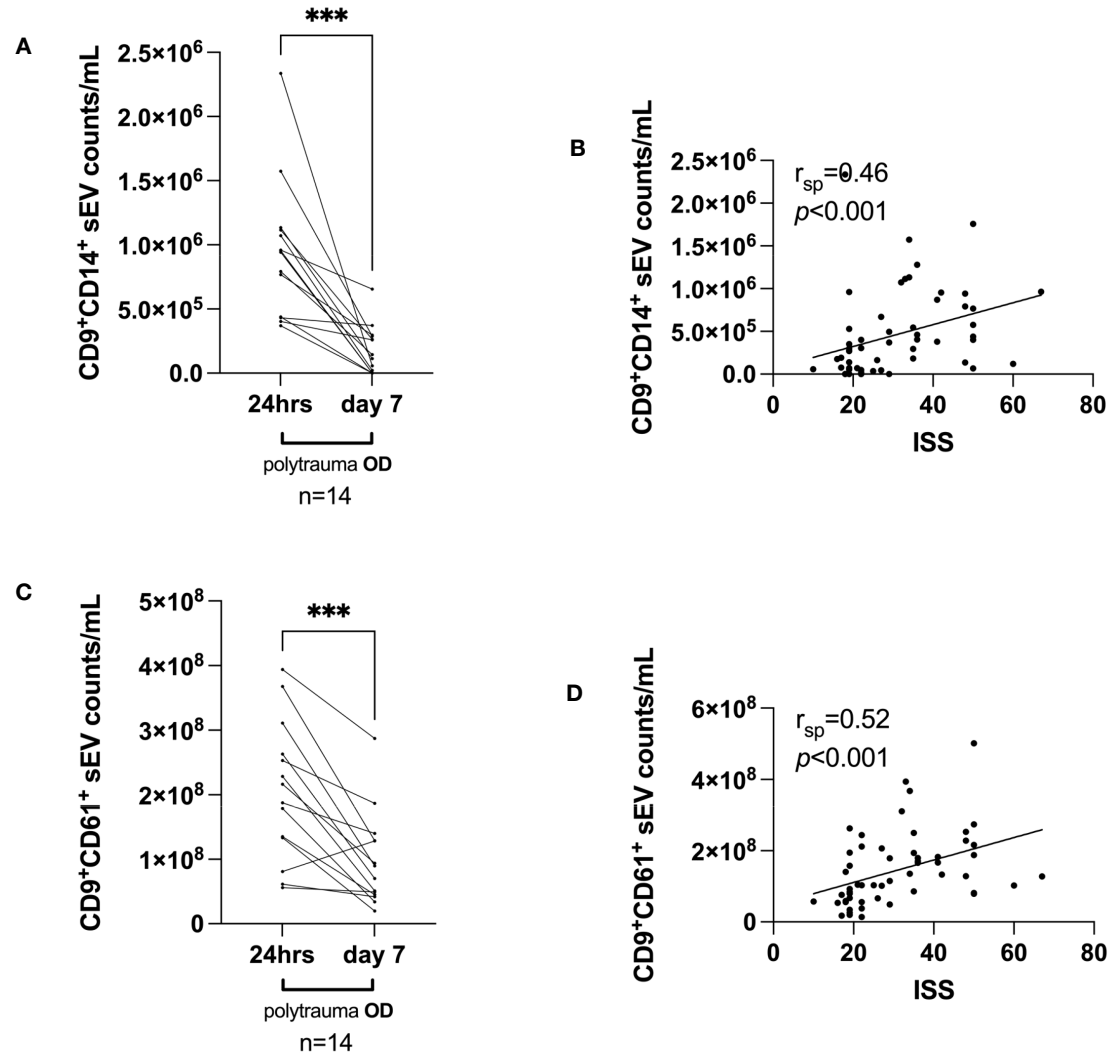
Small EV release on the investigated time axis (day 7 post-trauma) and correlations with ISS, days of invasive ventilation, and duration of ICU. **(A)** Median CD9^+^CD14^+^ small EV values and **(C)** median CD9^+^CD61^+^ small EV values within 24 h post-trauma *vs.* day 7 post-trauma in polytrauma OD patients, selected based on the availability of serum samples at day 7. Statistical significance was assessed by a two-tailed, unpaired t-test (*p < 0.05; **p < 0.01; ***p < 0.001; **(A)** t = 5.063, df = 13; **(C)** t = 4.783, df = 13). *Post-hoc* calculated power (1 − β err prob) is 1.0. **(B, D)** Spearman’s correlation (r_sp_) analyses for median CD9^+^CD14^+^ small EVs and CD9^+^CD61^+^ small EV values and associated ISS value (n = 56) as assessed 24 h post-trauma. *p* and r_sp_ values as indicated. EV, extracellular vesicle; ISS, injury severity score; ICU, intensive care unit.

Here, we randomly selected 14 polytrauma OD patients based on serum sample availability to be assessed on day 7 post-trauma. Overall, we observed that the median number of CD9^+^CD14^+^ small EVs dropped sharply on day 7 from a median of 9.4925 × 10^5^/mL serum (day 1) to a median of 1.3005 × 10^5^/mL serum (*p* = 0.0002) (**Figure 5A**). A similar decrease was observed from 24 h post-trauma to day 7 post-trauma for CD9^+^CD61^+^ small EVs from a median of 2.0203725 × 10^8^/mL serum to a median of 7.985925 × 10^7^/mL serum CD9^+^CD61^+^ small EVs (*p* = 0.0004) (**Figure 5C**).

Next, we studied whether the measured small EV subpopulations in polytrauma patients would correlate with ISS. Indeed, median CD9^+^CD14^+^ small EVs (**Figure 5B**) and CD9^+^CD61^+^ small EVs (**Figure 5D**) values isolated from serum taken within 24 h post-trauma significantly correlated with ISS (both *p* < 0.001) with r_sp_ values of 0.46 and 0.52 (n = 56), respectively.

Of note, the focus on different qualities and severity of trauma in our study raises the question of whether the presence of small EV subpopulations could predict disease outcomes. This could be measured as the duration of invasive ventilation and an intensive care unit (ICU) stay. **Supplementary Figures 5A, B** depict Spearman’s correlations of median CD9^+^CD14^+^ small EVs and median CD9^+^CD61^+^ small EVs as determined 24 h post-trauma with days of invasive ventilation. Median CD9^+^CD14^+^ small EVs correlated significantly with the duration of invasive ventilation (*p* = 0.003), whereas median CD9^+^CD14^+^ small EVs did not (*p* = 0.12). However, the r_sp_ values were below 0.5, 0.42 (**Supplementary Figure 5A**), and 0.23 (**Supplementary Figure 5B**). Therefore, they were not useful in the clinical setting and were neglected. Another critical patient parameter was the duration of ICU stay. Here, Spearman’s r_sp_ values were lower than 0.5: for CD9^+^CD14^+^ small EVs, r_sp_ = 0.47 (*p <*0.001, **Supplementary Figure 5C**), and for CD9^+^CD61^+^ small EVs, r_sp_ = 0.32 (*p* = 0.03, **Supplementary Figure 5D**).

### Alteration in small EV subpopulations distinguishes injured animals in a porcine model of polytrauma

**Figure 6:**
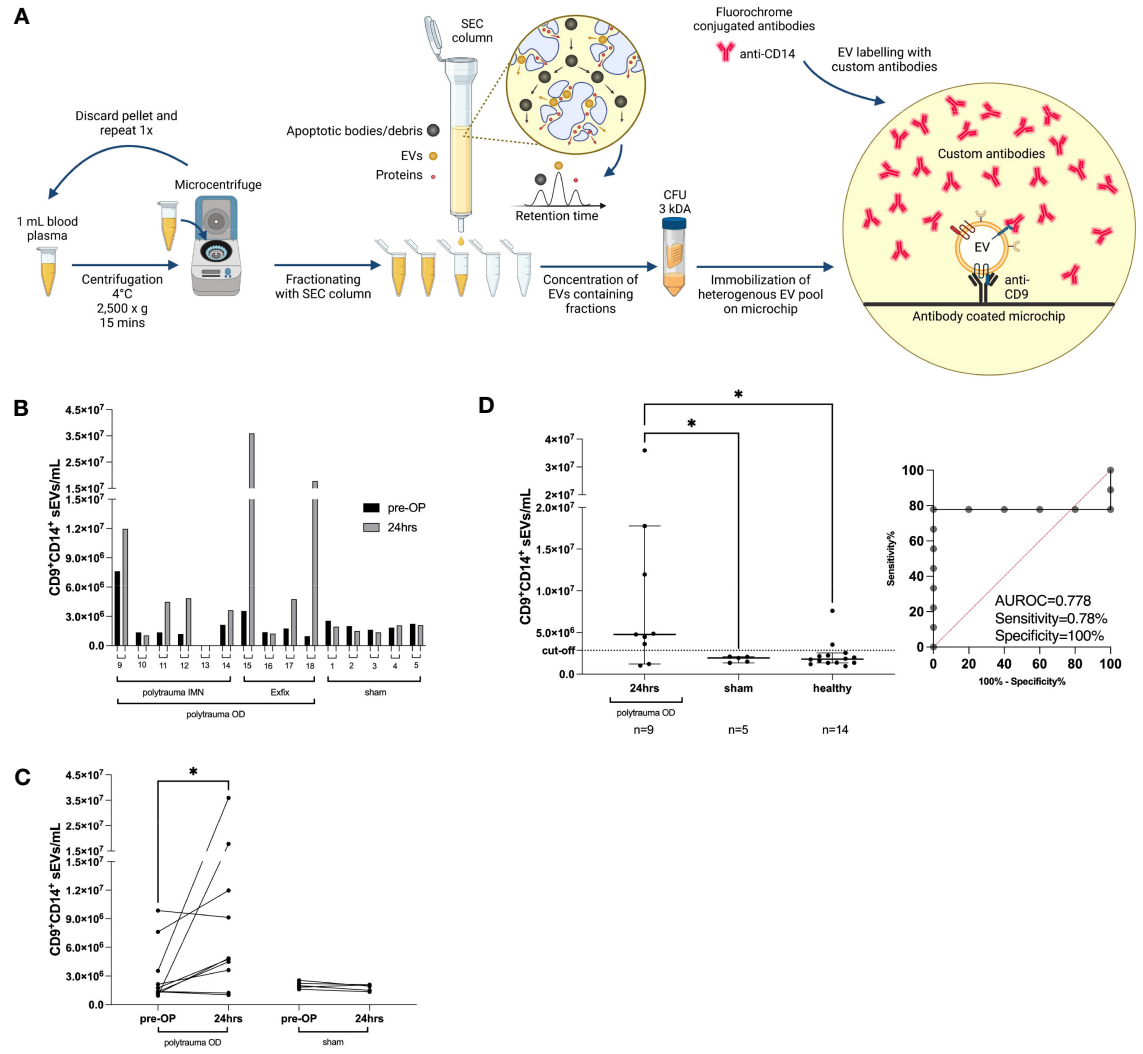
Porcine polytrauma model and CD9^+^CD14^+^ small EV performance before and after polytrauma induction. **(A)** Porcine plasma requires platelet removal by two 2,500 × *g* centrifugations prior to SEC-based EV purification, followed by UF of the first five fractions by 3-kDa MWCO centrifugation filters. The ExoFlex^®^ chip was coated with anti-porcine CD9 for the capture of CD9^+^ EVs. **(B)** Overview of individual CD9^+^CD14^+^ small EV values before and after trauma (24 h) induction for each pig, including the pig’s experimental number. Pigs are divided into three groups: polytrauma OD with external fixation (Exfix, n = 4), eight pigs with non-reaming intramedullary nailing (IMN), and the sham group (n = 5). **(C)** Overview of intragroup changes before (healthy) and after trauma induction (24 h) in the polytrauma OD and sham groups. **(D)** Median values of CD9^+^CD14^+^ EVs post-trauma in the polytrauma OD pigs compared to sham at 24 h and healthy pigs (defined as all pigs prior to polytrauma induction, baseline values; statistics assessed as follows: **(D)** one-way ANOVA including Fisher’s LSD *post-hoc* test for multiple comparisons, F = 3.959, df = 2; **(C)** two-tailed, unpaired t-test (*p < 0.05; **p < 0.01; ***p < 0.001; t = 1.928, df = 9). Note: CFU, centrifugal filter unit; EV, extracellular vesicle; SEC, size exclusion chromatography; UF, ultrafiltration; MWCO, molecular weight cut-off; OD, organ damage; LSD, least significant difference.

To evaluate whether the porcine polytrauma model would follow small EV distributions as in humans and serve for further evaluation of our study, we investigated 14 pigs before and after surgical induction of polytrauma, as depicted in **Figure 6A** and described in the Methods section in greater detail. Porcine ethylenediaminetetraacetic acid (EDTA) plasma was utilized as a small EV source before ExoFlex^®^ chip incubation (**Figure 6A**). Briefly, 16 experimental pigs were traumatized, eight pigs were stabilized with external fixation (Exfix), and eight pigs were stabilized with non-reaming intramedullary nailing (IMN). An additional eight experimental pigs underwent a control-sham operation. Owing to the restricted availability of ExoFlex^®^ chips and anti-porcine antibodies, 14 porcine samples were randomly measured (**Figure 6B**). Interestingly, an increase in CD9^+^CD14^+^ small EVs of 1.6-fold to 18.6-fold was detectable in seven out of nine polytrauma OD pigs compared to the median CD9^+^CD14^+^ small EV values prior to *vs.* post-polytrauma OD (**Figure 6C**). Small CD9^+^CD14^+^ EVs in the sham group remained stable at low levels.

The overall count of CD9^+^CD14^+^ small EVs in pigs with polytrauma OD was elevated compared to the sham-operated group (polytrauma OD 24 h: median = 4.762353 × 10^6^/mL plasma CD9^+^CD14^+^ small EVs, n = 9, *p* = 0.04; sham group 24 h: median = 1.945706 × 10^6^/mL plasma CD9^+^CD14^+^ small EVs, n = 5, *p* = 0.04) and compared to the healthy group (blood taken prior to operation: median = 1.799789 × 10^6^/mL plasma CD9^+^CD14^+^ small EVs, n = 14) (**Figure 6D**). One-way ANOVA with Fisher’s LSD test *post-hoc* test indicated a significant difference (*p* < 0.01) between the polytrauma OD (24 h) and sham groups (24 h) but no significance was found between the sham group (24 h) and healthy pigs prior to operation (**Figure 6D**). The calculated cut-off for CD9^+^CD14^+^ small EVs was 2.856334 × 10^6^/mL plasma, associated with an AUROC of 0.7778, a sensitivity of 78%, and a specificity of 100% (**Figure 6D**). The associated representative spot pictures are shown in **Supplementary Figure 5**.

## Discussion

The aim of our LiBOD pilot study was to investigate whether circulating blood-derived small EVs allow objective differentiation between polytrauma patients with and without internal organ damage. To this end, in our retrospective LiBOD pilot study, we offered insights into an objective assessment of polytrauma quality using a liquid biopsy and subsequent quantification of the indicated small EV subpopulations such as CD9^+^CD14^+^, CD9^+^CD61^+^ small EVs, and CD9^+^CD14^+^CD61^+^. These results could be crucial in practice to capture the quality of polytrauma, for example, when triage is required while medical resources, especially human resources such as paramedics, are scarce or insufficient (20, 21). In addition, overtriage and undertriage could be reduced or prevented. Overtriage may lead to a misperception of urgency and unnecessary consumption of scarce resources (32). Conversely, undertriage may lead to higher patient mortality but is more likely to result in inadequate medical care (33).

The ExoView^®^ Tetraspanin Chips that were used are capable of detecting and quantifying various subpopulations of small EVs derived from monocytes and platelets, including megakaryocytes. The specific antigen combinations used to define these subpopulations are as follows: for monocyte-derived subpopulations, CD9^+^CD14^+^, CD63^+^CD14^+^, CD81^+^CD14^+^, CD9^+^CD14^+^CD61^+^, CD63^+^CD14^+^CD61^+^, and CD81^+^CD14^+^CD61^+^; for platelet-, monocyte-, and megakaryocyte-derived small EVs, CD9^+^CD61^+^, CD63^+^CD61^+^, and CD81^+^CD61^+^. It is important to note that small EVs lacking expression of the three major tetraspanins, CD9, CD63, and CD81, were not immobilized on the ExoView^®^ Tetraspanin Chips (31). Therefore, the method we chose is similar to that of studies using anti-CD9, anti-CD63, and anti-CD81 beads to detect EV subpopulations (34). The advantage of the current measurement technique is its ability to stain the captured EVs on the chip simultaneously distinguish between subpopulations and determine their individual sizes. Thus, this technique allows single-EV resolution for these parameters.

Our analysis of the small EV subpopulations revealed that the CD9^+^CD14^+^ subpopulation of small EVs derived from monocytes appeared to be superior overall in accurately indicating polytrauma with organ damage, delivering sensitivity and specificity of 81% and 97%, respectively (**Figure 4A**), associated with an AUROC of >0.90 (**Figure 4A** and **Supplementary Table 5**). These values are more than acceptable in triage conditions and allow for prospective assessment of internal organ damage in blunt trauma in the absence of open wounds, which would clearly indicate internal organ injury. A typical scenario for undertriage can be fatal if internal injuries are not detected in a timely manner or at the first symptoms of unconsciousness due to internal bleeding/haemorrhagic shock (35).

Going in line with our monocyte small EV data, we detected significantly increased levels of CD9^+^CD61^+^ small EVs in the sera of patients with polytrauma OD (**Figures 4B, C**). These small EVs likely originated in part from subsets of monocytes and most likely from platelets and their precursors as megakaryocytes. Platelet activation is an early event in injury, and platelets release EVs (16, 36). However, CD61 alone does not pinpoint platelets as the source of CD9^+^CD61^+^ small EVs, which were inferior compared to monocyte-derived small EVs (**Supplementary Table 5**). Thus, CD9^+^CD61^+^ small EVs could likely be released by a novel subset of monocytes, as recently suggested by Hamers A.A.J. et al. based on their high-dimensional mass cytometric analyses of three human CD16^+^ non-classical monocyte subsets and four CD14^+^ classical monocyte subsets (37). The classical subset 1 appeared to have high CD61 and CD9 expression (37), making them also potential EV donors for CD9^+^CD14^+^CD61^+^ small EVs (**Figure 4C**).

We observed a marked and significant decrease in small CD9^+^CD14^+^ and CD9^+^CD61^+^ EVs 7 days post-trauma compared with 24-h values in patients who did not develop any post-trauma complications such as systemic inflammatory response syndrome (SIRS), acute respiratory distress syndrome (ARDS), or sepsis (38). Other studies have reported that CD14^+^ small EVs are elevated in ARDS (39). This rapid decrease contrasts with reports in which an increase in CD45^+^ large EVs was measured over time up to day 28 (40). This discrepancy could easily be explained by the fact that the authors measured CD45^+^ large EVs (40), CD14^+^ is only a small part of the CD45^+^ compartment, and large EVs might have different pharmacokinetic behaviours than small EVs *in vivo*. As described by Yáñez-Mó et al., the clearance time of EVs in circulation may depend on their steady-state level and the balance between the generation and clearance of each EV population (41, 42).

Next, we investigated whether correlations could be calculated between the measured small EV populations and the ISS. Our data showed that CD9^+^CD14^+^ small EVs and CD9^+^CD61^+^ small EVs were significantly correlated with ISS [Spearman’s r_sp_ = 0.46 (**Figure 5B**) and r_sp_ = 0.52 (**Figure 5D**)]. However, several variables need to be considered: ISS assessment is highly subjective and prone to observer error (43). In their study, the authors observed a probability of only 28% that the assessments of 16 trauma patients by 15 trauma and injury experts were consistent and a probability of 51% for assigning individual cases to the same severity group (43). This limits the validity of our correlations between ISS and reported subpopulations of small EVs. For large EVs, a correlation between ISS and CD61^+^ large EVs in trauma was reported, with an r_sp_ value of 0.635 (n = 22) (15). We calculated a ranked correlation for CD9^+^CD61^+^ small EVs with an r_sp_ value of 0.52 (**Figure 5D**), showing a similar trend.

As our LiBOD pilot study included only 61 severely injured patients (ISS ≥ 15), we chose a polytrauma animal model for independent validation. Here, we took advantage of an already established porcine trauma model (28, 29, 44) in which pigs received blunt chest trauma, bilateral femur fractures, and standardized liver laceration combined with pressure-controlled haemorrhagic shock (35 ± 5 mmHg) for 90 min, mimicking typical polytrauma (ISS ≥ 15) with internal organ damage (polytrauma OD). Our porcine polytrauma study data mirrored our human patient data, and CD9^+^CD14^+^ small EVs successfully separated injured animals from sham-operated animals and samples collected before trauma (**Figure 6D**). While we were able to successfully replicate our findings from the human study using a porcine trauma model, it is important to note that we intentionally chose porcine plasma as the source of small EVs instead of serum. The binding of small EVs to the thrombus when using serum as an EV source remains speculative and presents a potential systematic flaw in human samples. To mitigate any potential bias or artefacts associated with serum as an EV source, we opted to utilize plasma as the small EV source in our porcine model, thereby ensuring more reliable and accurate results.

Moreover, by using the porcine polytrauma model to validate our findings, we were able to confirm that the medication and drugs administered to human polytrauma patients, such as fibrinogen and tranexamic acid (among others, which can be found in **Supplementary Figure 1**), did not have a relevant impact on the release of EVs. We conducted Spearman’s correlation analysis to examine the potential relationship between the administered drugs and the release of EVs, as well as their influence on specific subpopulations of small EVs, including CD9^+^CD14^+^. The results of this analysis are summarized in **Supplementary Table 6**.

## Conclusion

Our LiBOD pilot study has to be evaluated in a larger cohort size with an increased diversity of traumas, including brain trauma, likely associated with undertriage (45). Desired sampling would be at the scene of the accident to best determine the trauma level as early as possible or during transport to a trauma centre providing an appropriate level of trauma care. Such early decision-making based on LiBOD could potentially not only avoid undertriage but also reduce cost per patient in cases of overtriage (32). Overall, a small EV assessment as part of LiBOD has the potential to assist subjective triage with an objected value to prevent undertriage and overtriage and to assist in providing the best and fastest medical care for trauma patients.

## Data availability statement

The raw data supporting the conclusions of this article will be made available by the authors, without undue reservation.

## Ethics statement

The studies involving humans were approved by the State Chambers of Medicine in Rhineland-Palatinate, Germany (ANr.:2020-15050). The studies were conducted in accordance with local legislation and institutional requirements. The participants provided their written informed consent to participate in this study. The animal study was approved by the State Agency for Nature, Environment, and Consumer Protection of North Rhine-Westphalia (Landesamt für Natur, Umwelt und Verbraucherschutz Nordrhein-Westfalen AZ:81-02.04.2020. A215).

## Author contributions

BW: Formal analysis, Investigation, Validation, Writing – original draft, Data curation. AW: Data curation, Investigation, Project administration, Resources, Writing – review & editing. JG: Data curation, Methodology, Project administration, Resources, Writing – original draft, Writing – review & editing. RJSS: Formal analysis, Methodology, Visualization, Writing – original draft, Writing – review & editing. CK: Investigation, Visualization, Writing – review & editing. TT: Investigation, Resources, Writing – original draft, Writing – review & editing. QZ: Investigation, Methodology, Resources, Writing – review & editing. ÜM: Investigation, Methodology, Resources, Writing – review & editing.. KH: Investigation, Methodology, Resources, Writing – review & editing. LL: Methodology, Resources, Writing – review & editing. MH-L: Investigation, Methodology, Resources, Writing – review & editing. MG: Investigation, Methodology, Resources, Writing – review & editing. TM: Investigation, Methodology, Resources, Writing – original draft, Writing – review & editing. SS: Data curation, Investigation, Methodology, Resources, Writing – original draft, Writing – review & editing. RS: Funding acquisition, Investigation, Methodology, Resources, Writing – original draft, Writing – review & editing. CS: Resources, Writing – review & editing. IS-W: Resources, Writing – review & editing. BG: Methodology, Resources, Validation, Writing – original draft, Writing – review & editing. FH: Investigation, Methodology, Resources, Writing – review & editing. VL-K: Conceptualization, Funding acquisition, Investigation, Methodology, Project administration, Resources, Supervision, Validation, Writing – original draft, Writing – review & editing. AGW: Conceptualization, Data curation, Funding acquisition, Investigation, Project administration, Resources, Supervision, Writing – original draft, Writing – review & editing. MTK: Conceptualization, Formal analysis, Funding acquisition, Investigation, Methodology, Project administration, Resources, Supervision, Validation, Visualization, Writing – original draft, Writing – review & editing.
