# Supplementary material for: Liquid Biopsy in Organ Damage: small extracellular vesicle chip-based assessment of polytrauma
Source: Front Immunol. 2023 Nov 15;14:1279496. doi: 10.3389/fimmu.2023.1279496 (PMC10684673; doi:10.3389/fimmu.2023.1279496)
Supplement: Supplementary file 1 [file DataSheet_1.pdf]

LiBOD – Liquid Biopsy in Organ Damage – small extracellular vesicle chip-based assessment of polytrauma

Bingduo Wang[1], Aliona Wöhler[2], Johannes Greven[3], Rebekka J. S. Salzmann[1], Cindy M. Keller[1], Tobias Tertel[4], Qun Zhao[3], Ümit Mert[3], Klemens Horst[3], Ludmila Lupu[5], Markus Huber-Lang[5], Martijn van Griensven[6], Tom Erik Mollnes[7,8,9], Sebastian Schaaf[2], Robert Schwab[2], Christian P. Strassburg[1], Ingo G. H. Schmidt-Wolf[10], Bernd Giebel[4], Frank Hildebrand[3], #Veronika Lukacs-Kornek[11], #Arnulf G. Willms[11,12] and #Miroslaw T. Kornek[1,2]

#contributed equally

Supplement Material and Methods

Human cell lines

The THP-1 cell line was obtained from DSMZ (Leibniz Institute DSMZ-German Collection of Microorganisms and Cell Cultures, Braunschweig, Germany, ACC16). Cells were grown in RPMI-1640 GlutaMAX medium (Gibco by Life Technologies, Paisley, UK) with 10% h.i. FBS (Gibco by Life Technologies, Paisley, UK) and 1% penicillin-streptomycin solution (10,000 U/mL, Gibco by Life Technologies, Paisley, UK).

Differentiation and stimulation of THP-1 monocytes cells in vitro

THP-1 cells were differentiated into macrophage-like cells with phorbol 12-myristate-13-acetate (PMA) (Sigma Aldrich, Missouri, USA; P1585) or into lipopolysaccharide (LPS)-induced LPS-induced macrophages (Merck, Darmstadt, Germany, L3129) from *Escherichia coli*. 5.0 × 10^5 THP-1 cells/mL were resuspended in growth medium and cells were incubated with 200 nM PMA at 37°C, 5% CO_2 for 72 hrs. The PMA-containing medium was removed, and macrophage-like cells were washed with PBS (pH 7.4) (Gibco by Life Technologies, Paisley, UK). Macrophage-like cells were maintained in growth medium for 5 days. To further stimulate cells into LPS-induced macrophages, the cells were kept in growth medium overnight, washed with PBS (pH 7.4), and further stimulated with 500 ng/mL LPS for 3 hrs (1). The LPS-containing medium was removed, and cells were kept in fresh growth medium for an additional 72 hrs. In the case of PMA-only differentiation, cells were washed after PMA treatment and maintained in fresh medium for an additional 5 days. The correct phenotype was confirmed by cell surface staining and subsequent fluorescence-activated cell sorting (FACS).

Cell surface staining for flow cytometric analysis of THP-1 human cell line

Per staining 1.5 x 10^5 cells were resuspended in 50 µL FcR blocking reagent (Miltenyi Biotec, Bergisch Gladbach, Germany) diluted in FACS buffer (1x PBS, 5 % BSA, 2 mM EDTA, and 0.1% sodium azide) 1:20 (v/v) and incubated for 5 min in darkness on ice. The respective antibodies or isotype mix (Suppl. Table

2) were added, and cells were incubated for an additional 15 min in the dark on ice. Designated antibodies were added according to the manufacturer's recommendations and titrated against their matching isotype controls (Suppl. Table 2). The cells were then incubated in the dark on ice for 15 min. Subsequently, 3 µL of 7-AAD (BD Pharmingen, New Jersey, USA, 559925) was added and cells were incubated in the dark at 4°C for another 5 min. The cells were analyzed on a BD FACSCanto II flow cytometer (BD Biosciences, New Jersey, USA). All experiments were performed in technical and biological triplicates.

Isolation and purification of THP-1 derived small EVs by size-exclusion chromatography (SEC) and ultra-filtration (UF) from cell culture supernatant

When cells have reached at least 50% confluence, cell culture supernatants were harvested and centrifuged at 500 × g for 10 min to remove cells, cell debris, and larger apoptotic bodies. This was followed by centrifugation at 10,000 × g for 10 min to remove the large EVs. The Large EV depleted supernatant was then transferred to a 100 kDa MWCO filter (Amicon Ultra, Merck, Darmstadt, Germany) and concentrated to a volume of 2 mL at room temperature (RT). The sample was loaded onto a qEV2/35 SEC column (Izon Science, Christchurch, New Zealand), and the buffer flow volume of 14.1 mL was discarded, after which the following five fractions (2 mL each) were collected. The collected fractions were concentrated to 1 mL using a 3 kDa MWCO filter (Amicon Ultra, Merck, Darmstadt, Germany) and stored at -80°C until further use.

Isolation of THP-1 derived large EVs by centrifugation from cell culture supernatant

Large EVs were isolated according to previously pre-established protocols (2-4). The supernatants of LPS-induced macrophages were centrifuged at 300 × g for 5 min at 4 °C to remove cells, cell debris, and larger apoptotic bodies. The supernatant was further purified by centrifugation at 2,000 x g for 30 min, followed by centrifugation at 20,000 × g for 1 h at 4 °C. Resulting large EV-enriched pellets were resuspended in filtered PBS, pH 7.4 (0.22 µm) (Merck Millipore, Boston, USA) and stored at -80℃ until usage.

Isolation of serum from human blood

Blood sampling was carried out in accordance with the recommendations of the European Liquid Biopsy Society (ELBS) and followed the ISO 20186-1:2019 '*Molecular in vitro diagnostic examinations — Specifications for pre-examination processes for venous whole blood*. ' ISO 20186 contains specific guidelines on the processing of venous blood and small EVs. Serum samples were obtained from polytrauma patients with an ISS ≥ 15. 10 mL of whole blood was collected from a venous or arterial access post-traumatic. Blood samples were collected within 24 hrs and 7 days post-trauma. Full-blood was centrifuged at 3000 × g for 3 min at RT, and the supernatants were collected. Sera were stored permanently at -80 °C. For the shipment of human specimens, the following regulations were strictly obeyed as UN 2814, UN 3291, or UN 3373 (UN Recommendations on the Transport of Dangerous Goods) without interrupting the freezing chain.

Transmission electron microscopy of EVs

The isolated EV samples were diluted 100-fold with ultrapure water. 7.5 µL of the sample solution was mixed with 1 µL of aqueous contrast solution containing 1% methyl cellulose (w/v; Sigma Aldrich, Darmstadt, Germany) and 2% uranyl acetate (w/v; Polysciences, Warrington, USA). After incubation for 10 min, a 0.5 µL droplet was placed on a 200-mesh copper grid covered with a carbon-coated Formvar film (Plano, Wetzlar, Germany) and dried at room temperature to allow EVs to adhere to the film surface. Images were taken using a JEM 1400Plus electron microscope (JEOL, Tokyo, Japan) at 120 kV, equipped with a 4096 × 4096 pixel CMOS camera (TemCam-F416; TVIPS, Gauting, Germany). The EMMENU image acquisition software (version 4.09.83) was used to obtain 16-bit images.

Protein isolation and quantification from small EVs

Small and large EVs were lysed 1:1 (v/v) in RIPA buffer (50 mM Tris-HCl, PH 8.8; 150 mM NaCl; 1 % NP-40; 0.5 % Sodium Deoxycholate; 0.1 % SDS; ThermoFisher, Waltham, USA) with Protease-Phosphatase-Inhibitor (ThermoFisher, Waltham, USA) (1∶1000). Lysis was followed by centrifugation at 11,000 g for 30 min at 4 °C. Protein concentrations were determined using the QubitTM Protein Broad Range Assay Kit (ThermoFisher, Waltham, USA) according to the manufacturer’s instructions.

Western blot analysis

Lysed samples were treated with the reducing agent dithiothreitol (DTT) to resolve the proteins according to their molecular weight. ProteinSimple capillary immunoassay (Wes) (ProteinSimple, Bio-Techne, Minneapolis, MN, USA) was performed with 2 µg of protein per capillary according to the manufacturer's instructions (5). The following primary antibodies were used for WES, as summarized in Suppl. Table 3.

Nanoparticle tracking analysis of small EVs

The size and concentration of isolated small EVs were measured by nanoparticle tracking analysis (NTA) using ZetaView S/N 17-333 (Particle Metrix, Meerbusch, Germany). Prior to the measurements, EVs were diluted in EV-free, filtered PBS (0.22 µm) to a final volume of 1 mL. Sample measurements were performed twice at 11 different positions. Standard operating procedures were set to camera control-sensitivity:79.2, frame rate:30, shutter:70; post-acquisition parameters - min brightness,30; max size,1000; min size,5; trace-length,15; nm/class,30; classes/decade,64.

Multiple trauma model – porcine study

a) Instrumentation and Anaesthesia

The porcine model has been described in details elsewhere (6-8). Therefore, only a short summary of the model is elucidated within this publication.

Before start of experiment, pigs completed a 12 hrs fasting period, with water *ad libitum*. On the day of operation, premedication (azaperone four mg/kg body weight) was administered by an intramuscular injection. During operation and observation time anaesthesia was maintained through continuous 1% propofol (Fresenius, Germany), midazolam (Panpharma, Germany) and fentanyl (Panpharma, Germany) administration. Ventilation with lung protective parameters during observation period (8 - 12 mL/kg/body weight, 50% oxygen) was carried out by usage of a Draeger Evita (Lübeck, Germany) ventilation device.

In order to maintain physiological homeostasis and nutrition in pigs continous aminoven (370 kcal/L 50 - 70 mL/kg body weight and day) and crystalloid infusions (Sterofundin ISO®; 0.5 - 2.0 mL/kg/hrs) were administered. These fluids and medication were administered through two central venous catheters (Arrow Medical, UK) placed into right femoral vein and external jugular vein. Mean arterial blood pressure (MAP) was measured *via* an arterial line (Vygon, Germany) placed into left femoral artery and monitored *via* a Philips patient monitor (Philips Health Systems, Hamburg, Germany). Finally, a suprapubic catheter was placed into the bladder. Pigs were warmed with air blowers to a body temperature of 38.5 – 39.5°C.

b) Trauma and Haemorrhage

After instrumentation, stable baseline conditions were reached after 120 min. Subsequently, oxygen supply was set to 21%, warming and previous continued fluid administration was put to 0.1 mL/hrs to keep the transfusion lines open but not to provide further fluid supply and trauma was induced and maintained for 90 min (trauma phase).

During trauma phase, polytrauma pigs (PT) received a blunt chest trauma with a bolt gun (Blitz-Kerner, turbocut JOBB GmbH, Germany) fired at a double layer panel of 1 cm lead on top and 1 cm steel. The same device was used to induce femur fractures on both sides using a T-shaped steel panel. Furthermore, a midline mini-laparotomy was performed to explore right hepatic lobe. Standardized liver laceration in combination with pressure controlled haemorrhagic shock (40±5 mm HG) were induced for 90 min *via* blood drawing through venous catheter.

After trauma phase resuscitation of pigs including reperfusion of blood and fluids, FiO_2 levels of 50 %, warming and administration of antibiotics (Ceftriaxon® 2 g i.v./every 24 hrs) were performed regarding established trauma guidelines (ATLS®, AWMF-S3 guideline on Treatment of Patients with Severe and Multiple Injuries®). The resuscitation phase was followed by surgical phase (OP phase; 60 min).

c) Group allocation

Altogether, 8 pigs were stabilized with external fixation (Radiolucent Fixator, Orthofix), and 8 pigs with non-reaming intra medullary nailing (T2 System, Stryker) following the DCO and ETC guideline procedures. 6 pigs were allocated to the Sham group; therefore, no intervention other than general instrumentation and anaesthesia was performed.

Plasma isolation from full-blood – porcine study

The EDTA K blood plasma samples (Sarstedt, Nümbrecht, Germany, S-Monovette) were centrifuged at 2000 g for 15 min at 4 °C immediately after blood draw within the operation theatre to separate blood cells from plasma. Subsequently, plasma was filtered through a 0.22 µm syringe filter (Sartorius, Göttingen, Germany Cat# 16532) to remove cell debris. Plasma was stored at -80˚C for further use, and no additional melting or freezing cycles were added prior to EV isolation.

Supplement Figures and Tables

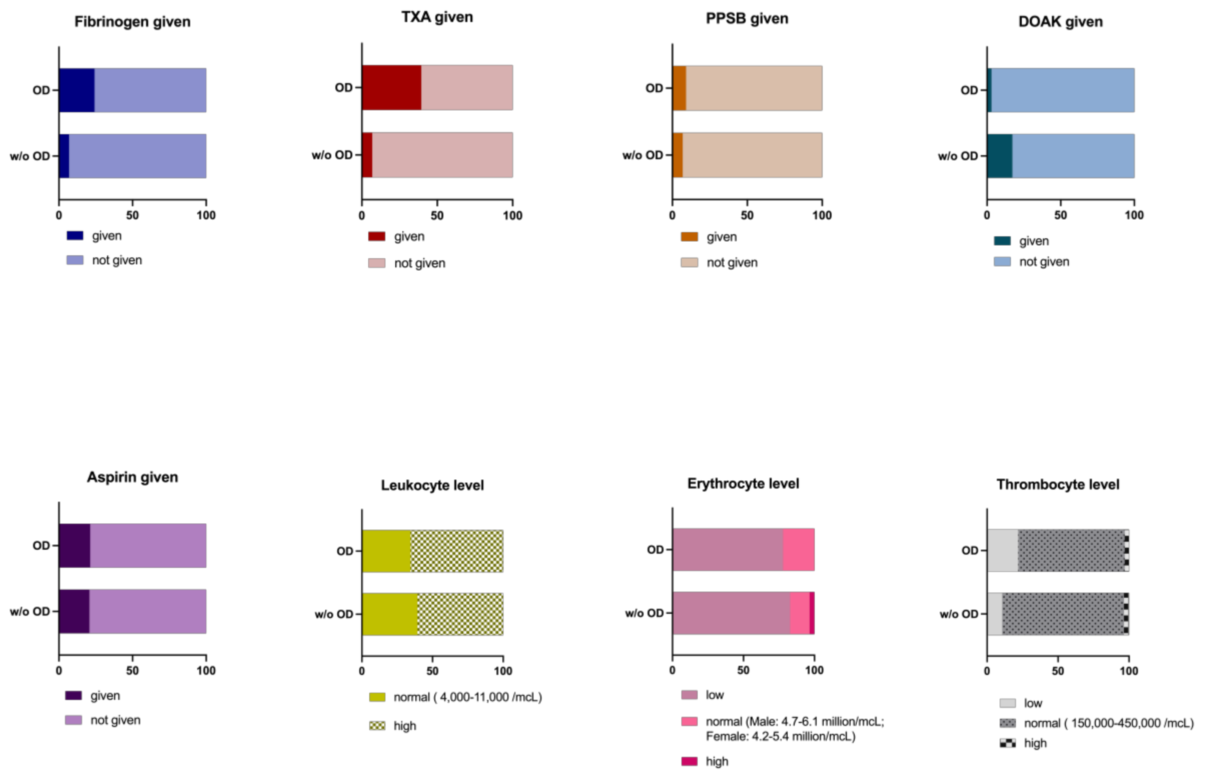

Suppl. Figure 1: Extended Polytrauma (ISS ≥ 15) Patient's demographics. Overview of given medications/drugs as indicted including pre-medication taken by patients on a daily base pre-trauma as Aspirin and overview of blood parameters as indicated. Correlation calculation between depicted parameters and their use is depicted in Suppl. Table 2.

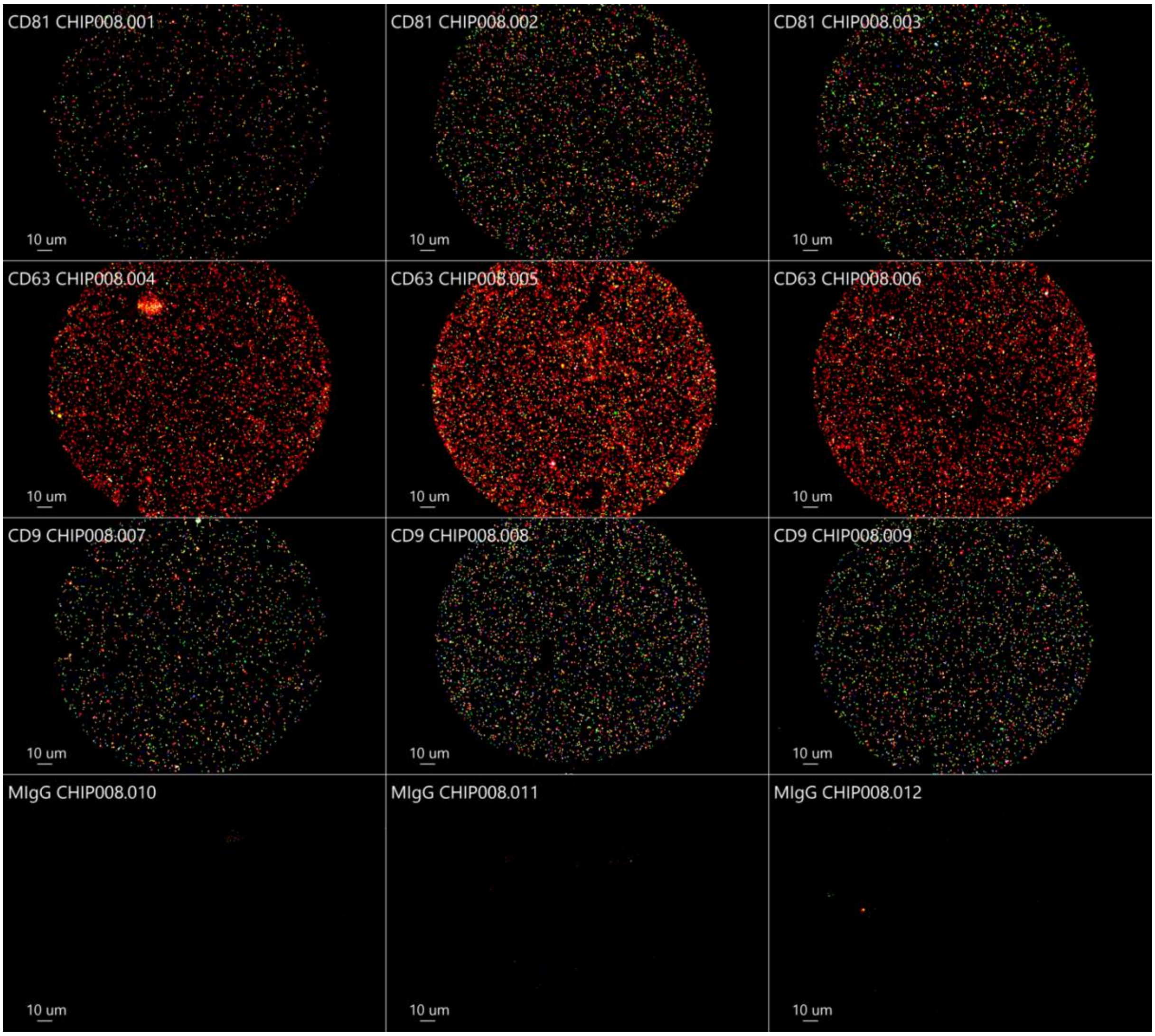

Suppl. Figure 2: Representative ExoView® Tetraspanin spots – cell culture derived small EVs. Depicted are exemplary original composite color dot spot images for tetraspanins CD9^{+} (blue) and CD63^{+} (red) and CD81^{+} (green) small EVs purified from culture as captured by anti-CD9, anti-CD63 and anti-CD81 capture spots on ExoView® Tetraspanin Chips with lab internal number 008 and corresponding isotype MIgG composite image as control for unspecific small EV capture. Depicted are all spots as measured by ExoView® Reader R100 which are selected and analyzed by ExoView® Analysis software automatically.

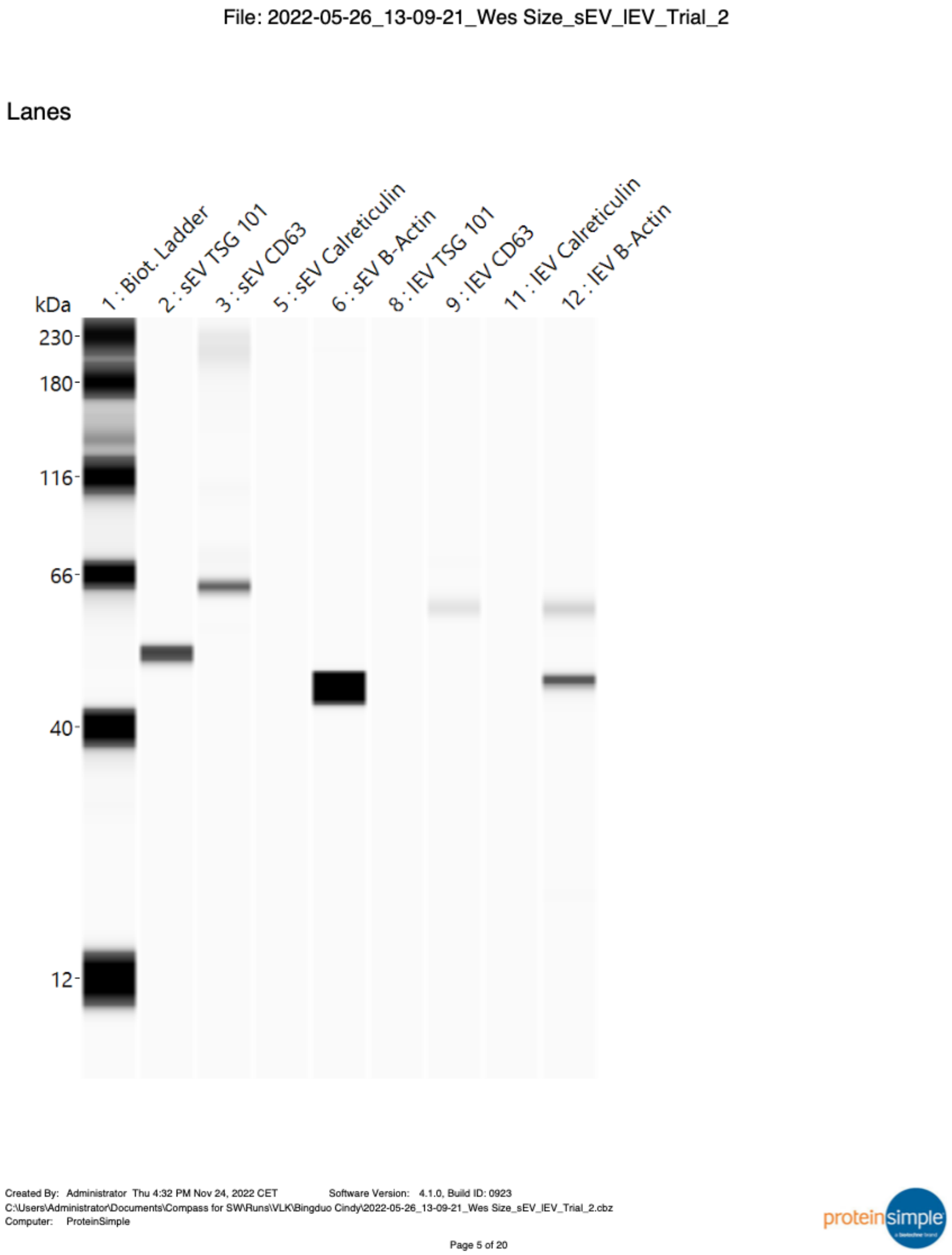

Suppl. Figure 3: Representative ProteinSimple capillary immunoassay (Wes) picture of purified small EVs. Immunoblot of 2 µg EV protein per capillary according to manufacturer's instructions for indicated small and large EV markers according to MISEV2018 guidelines. Depicted is page 5 from original WES report as provided by automated WES analysis software. Full report is attached as separated supplement file in original. TSG 101: tumor susceptibility gene 101; CD63: also known as Tetraspanin-30.

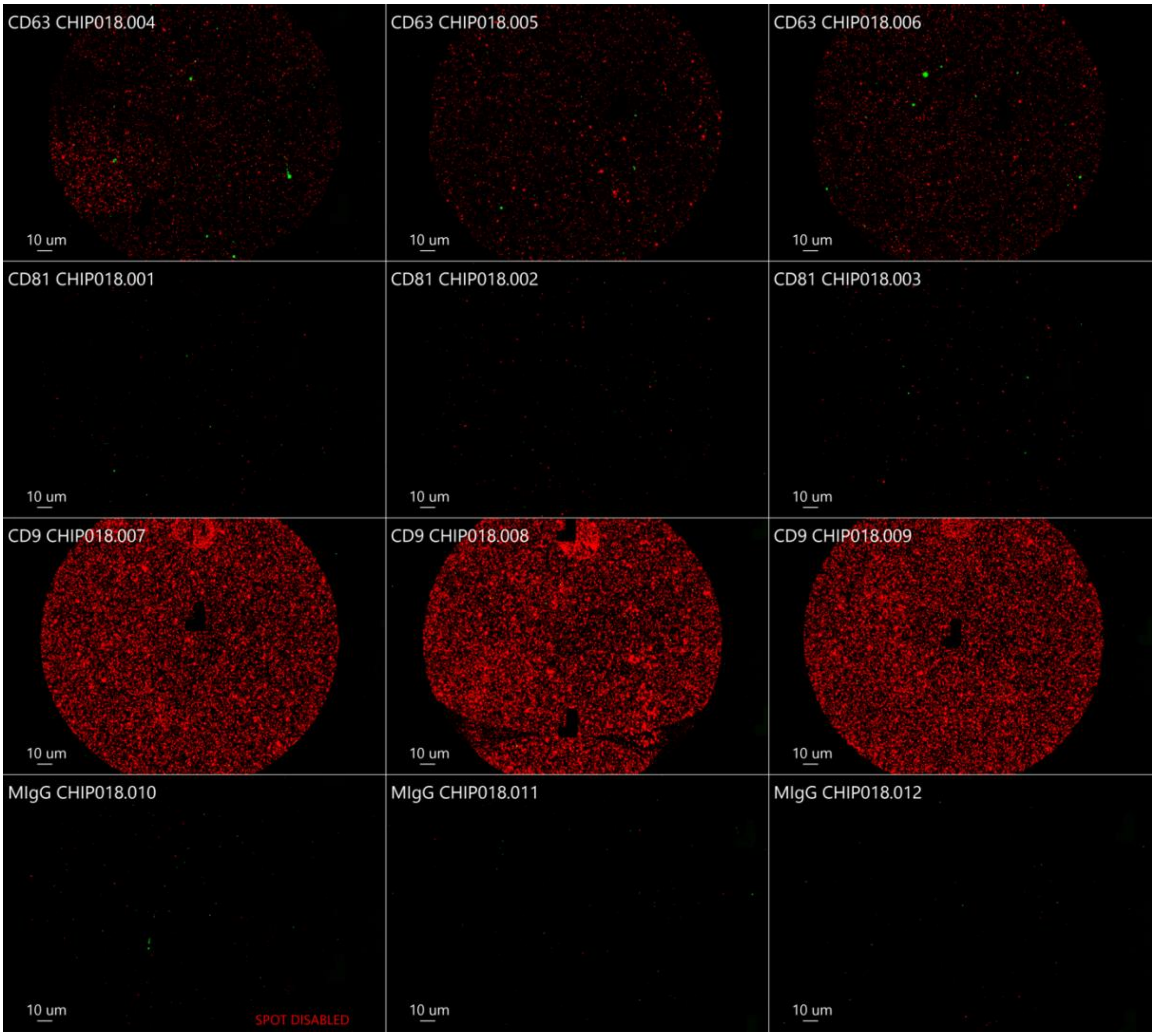

Suppl. Figure 4: Representative ExoView® Tetraspanin spots – human serum derived small EVs. Depicted are exemplary original composite colour dot spot images for $CD14^+$ (green) and $CD61^+$ (red) and $CD14^+CD61^+$ (yellow) small EVs purified from human serum as captured by anti-CD9, anti-CD63 and anti-CD81 capture spots on ExoView® Tetraspanin Chips with lab internal number 018 and corresponding isotype MIgG composite image as control for unspecific small EV capture. Depicted are all spots as measured by ExoView® Reader R100 which are selected and analysed by ExoView® Analysis software. MIgG CHIP018.010 was disabled by analysis software and designated as SPOT DISABLED in red.

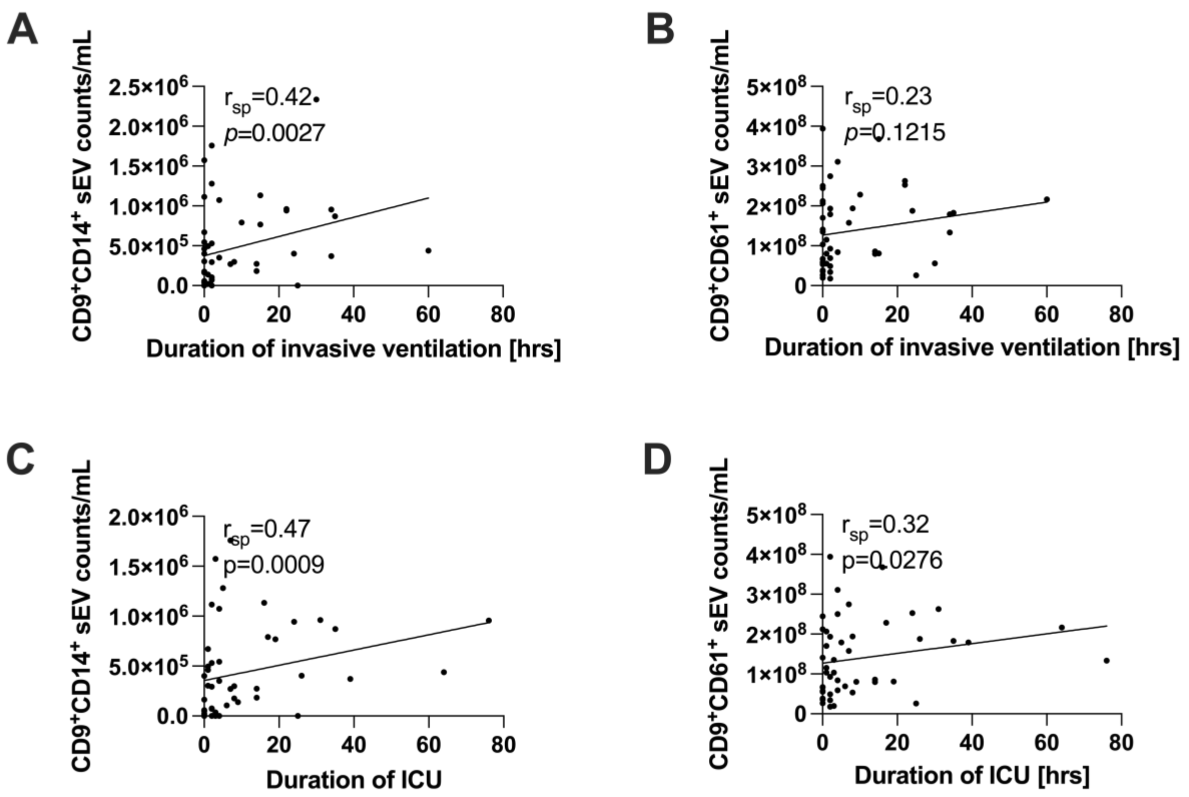

Suppl. Figure 5: small EV release on investigated time axis (day 7 post trauma) and correlations with days of invasive ventilation and duration of ICU. (A) and (B) depicts Spearman correlation analysis for median $CD9^+CD14^+$ small EVs and $CD9^+CD61^+$ small EV values with associated patient's days of invasive ventilation (n = 48). (C) Spearman correlation analysis for median $CD9^+CD14^+$ small EVs values and (D) $CD9^+CD61^+$ small EV values associated with patients' ICU duration (n = 47). *P* – and r_{sp} values as indicated.

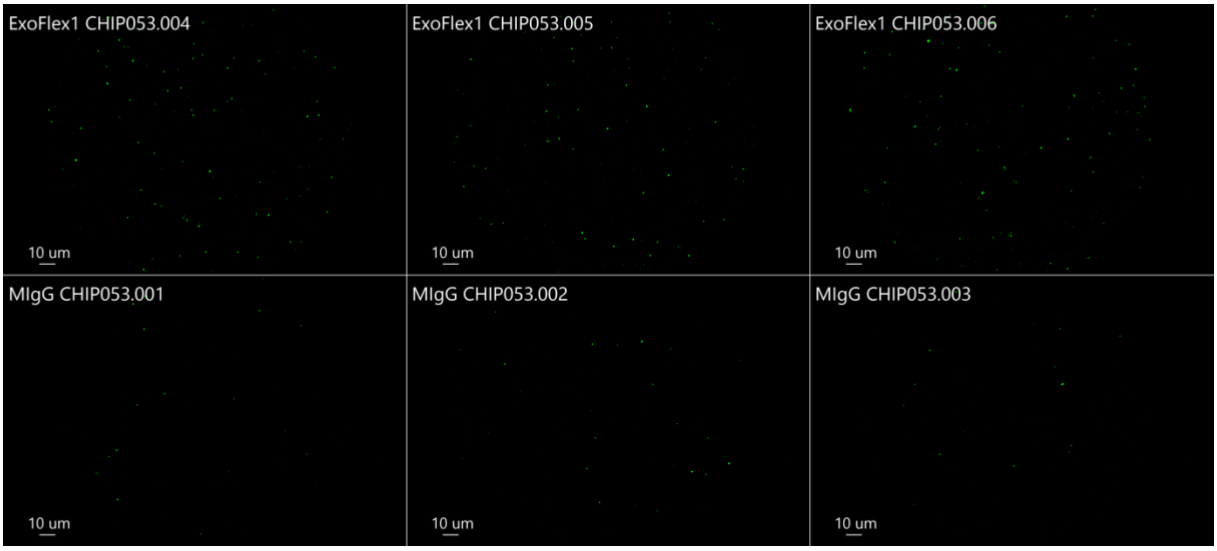

Suppl. Figure 6: Representative ExoFlex® Tetraspanin spots – porcine plasma derived small EVs. Exemplary original color dot spot images are shown for CD14+ (in green) particles isolated by SEC and UF from porcine plasma as captured by anti-CD9 and stained for CD14. MIgG as unspecific small EV binding control. Depicted are all spots on ExoFlex® chip with lab internal number 53 as measured by ExoView® Reader R100 which are analyzed by ExoView® Analysis software.

Supplement Tables

Suppl. Table 1 Patients' demographics

Polytrauma OD: n=32, w/o OD: n=29	Age, median [years]	Male gender [%]	Female gender [%]	ISS, median	Days in ICU, median [days]	Duration of invasive ventilation [days]	ASA [%]		
							I	II	III
OD	51.5	73.1	26.9	36	7.5	8	8	60	32
w/o OD	58	80	20	19	2	0	0	70.8	29.2

Polytrauma OD: n=32, w/o OD: n=29	Thoracic / abdominal organ damage [%]							Complications [%]					
	B	D	Bl	L	SP	LP	R	A	De	Dt	G	Po	Pn
OD	100	3.2	3.2	19.4	51.6	64.5	12.9	16	4	0	8	8	12
w/o OD	100	—	—	—	—	—	—	4.2	4.2	4.2	4.2	4.2	12.5

OD: organ damage; w/o OD: without organ damage; B: bone fracture; D: diaphragmatic rupture; Bl: bladder rupture; L: liver rupture; SP: spleen rupture; LP: lung contusion/ pneumothorax; R: renal rupture/ contusion; A: acute renal failure/ UTI; De: delirium; Dt: death; G: gastroparesis/GI bleeding; Po: post-trauma myocardial infarction/myocarditis/heart failure; Pn: pneumonia/ ARDS; note: some patient's had multiple organ traumas as L & LP or as SP & LP.

Suppl. Table 2 Overview of antibodies used for FACS analysis. Designated antibodies were added according to manufacturer's recommendations and antibodies were titrated against their matching isotype controls prior use.

Antibody (anti-human)	Conjugate	Catalog#	Vendor	Conc. [µg/µl]	Dilution
CD14	APC	170-078-099	Miltenyi Biotec	0.01	1:50
CD61	PE	130-110-749	Miltenyi Biotec	0.2	1:50
Isotype REA	APC	130-104-614	Miltenyi Biotec	0.02	1:100
Isotype REA	PE	130-113-341	Miltenyi Biotec	0.2	1:50

Suppl. Table 3 Overview of antibodies used for Western blot

Primary antibody	*Host/Isotype*	Catalog#	*Vendor*	*Dilution*
anti-TSG101	Rabbit IgG	ab125011	abcam	1:1000
anti-Calreticulin	Rabbit IgG	2891S	CellSignaling	1:1000
anti-CD63	Rabbit IgG	ab68418	abcam	1:300
anti-CD9	Rabbit IgG	PA5-85955	thermoFisher	1:1300
anti-β-actin	Mouse IgG_{2b}	926-42212	Li-Cor	1:100

Suppl. Table 4 Overview of antibodies for ExoView® Reader R100

Antibody	*Conjugate*	*Catalog#*	*Vendor*	*Conc. [µg/µl]*	*Dilution*
CD14 (anti-human)	APC	170-078-099	Miltenyi Biotec	0.01	1:10
CD61(anti-human)	PE	130-110-749	Miltenyi Biotec	0.2	1:200
CD14 (anti-pig)	FITC	MA5-28286	Invitrogen	0.1	1:100

Suppl. Table 5 Summarized data values of individual small EV populations in polytrauma including associated SEM, fold change and statistics

small EV	Median Polytrauma OD	SEM	Median Polytrauma w/o OD	SEM	Fold	*p*-value	cut-off	AUROC (*p* -value)	95% CI	sensitivity [%]	specificity [%]
$CD9^{+}CD14^{+}$	537200	89149	48000	22521	11.19	<0.001	360000	0.9461 (<0.001)	0.8953 to 0.9969	81.25	96.55
$CD9^{+}CD61^{+}$	174737500	18105443	67493500	10118299	2.59	<0.001	105860505	0.8599 (<0.001)	0.7671 to 0.9527	78.13	86.21
$CD9^{+}CD14^{+}CD61^{+}$	115250	22348	40000	8507	2.88	<0.001	87000	0.8699 (<0.001)	0.7578 to 0.9820	86.36	86.21
$CD63^{+}CD14^{+}$	20620	89472	0	19108		0.10	9250	0.6121 (0.13)	0.4706 to 0.7536	65.52	53.13
$CD63^{+}CD61^{+}$	55472000	13157614	21840000	4749709	2.54	<0.001	35459500	0.8351 (<0.001)	0.7312 to 0.9390	81.25	79.31
$CD63^{+}CD14^{+}CD61^{+}$	45600	10607	16500	6227	2.76	0.06	35000	0.6390 (0.06)	0.4991 to 0.7789	68.97	59.38
$CD81^{+}CD14^{+}$	0	9056	0	1138		0.41		0.5313 (0.68)	0.3857 to 0.6768		
$CD81^{+}CD61^{+}$	1485000	764728	715000	310254	2.08	0.24	1122108	0.5873 (0.24)	0.4433 to 0.7313	58.62	59.38
$CD81^{+}CD14^{+}CD61^{+}$	0		0								

Suppl. Table 6 Overview of calculated Spearman correlation between indicated drugs and blood parameters as indicated of the polytrauma patients with and without organ damage and associated small EVs numbers isolated by SEC and filtration from 1mL serum. Median EV count in polytrauma patients: 2.65 x 10^{11}EVs/mL Serum; median $CD9^+CD14^+$ small EV count in polytrauma: 3.0185 x 10^5/mL serum; median $CD9^+CD61^+$ small EV count in polytrauma: 1.2138 x 10^8/mL serum and median $CD9^+CD14^+CD61^+$ small EV count in polytrauma: 3.0185 x 10^5/mL serum. ns = not significant (p>0.05), r_{Sp} = Spearman correlation, n = 55 data pairs.

Medication/Drug given	r_{Sp}-value (signifc.) for mean EV	r_{Sp}-value (signifc.) for $CD9^+CD14^+$ small EVs	r_{Sp}-value (signifc.) $CD9^+CD61^+$ small EVs	r_{Sp}-value (signifc.) for $CD9^+CD14^+CD61^+$ small EVs
Fibrinogen	0.2585 (ns)	0.4153 (0.0016)	0.3498 (0.0088)	0.2338 (ns)
Tranexamic acid (TXA)	0.3407 (0.0102)	0.4418 (0.0007)	**0.4899 (0.0001)**	**0.4952 (0.0001)**
prothrombin concentrate (PPSB)	-0.0441 (ns)	0.1655 (ns)	0.1249 (ns)	0.1435 (ns)
DoAK new direct oral anticoagulants (DoAK)	-0.1360 (ns)	-0.2832 (0.0362)	-0.2755 (0.0417)	0.0349 (ns)
Aspirin	-0.0181 (ns)	0.0231 (ns)	-0.0058 (ns)	-0.1587 (ns)
Leucocyte level	0.0848 (ns)	0.1787 (ns)	0.0661 (ns)	0.0211 (ns)
Erythrocyte level	0.05486 (ns)	-0.0048 (ns)	0.0486 (ns)	0.0387 (ns)
Thrombocyte level	-0.1445 (ns)	-0.0321 (ns)	0.1179 (ns)	0.0722 (ns)

Supplement References

1. Daigneault M, Preston JA, Marriott HM, Whyte MK, Dockrell DH. The identification of markers of macrophage differentiation in PMA-stimulated THP-1 cells and monocyte-derived macrophages. PLoS One. 2010;5(1):e8668.
2. Julich-Haertel H, Urban SK, Krawczyk M, Willms A, Jankowski K, Patkowski W, et al. Cancer-associated circulating large extracellular vesicles in cholangiocarcinoma and hepatocellular carcinoma. J Hepatol. 2017;67(2):282-92.
3. Urban SK, Sanger H, Krawczyk M, Julich-Haertel H, Willms A, Ligocka J, et al. Synergistic effects of extracellular vesicle phenotyping and AFP in hepatobiliary cancer differentiation. Liver Int. 2020;40(12):3103-16.
4. Lukacs-Kornek V, Julich-Haertel H, Urban SK, Kornek M. Multi-Surface Antigen Staining of Larger Extracellular Vesicles. Methods Mol Biol. 2017;1660:201-8.
5. Budden CF, Gearing LJ, Kaiser R, Standke L, Hertzog PJ, Latz E. Inflammasome-induced extracellular vesicles harbour distinct RNA signatures and alter bystander macrophage responses. J Extracell Vesicles. 2021;10(10):e12127.
6. Horst K, Eschbach D, Pfeifer R, Hubenthal S, Sassen M, Steinfeldt T, et al. Local inflammation in fracture hematoma: results from a combined trauma model in pigs. Mediators Inflamm. 2015;2015:126060.
7. Horst K, Simon TP, Pfeifer R, Teuben M, Almahmoud K, Zhi Q, et al. Characterization of blunt chest trauma in a long-term porcine model of severe multiple trauma. Sci Rep. 2016;6:39659.
8. Lupu L, Horst K, Greven J, Mert U, Ludviksen JAK, Pettersen K, et al. Simultaneous C5 and CD14 inhibition limits inflammation and organ dysfunction in pig polytrauma. Front Immunol. 2022;13:952267.
